# Ercc2/Xpd deficiency results in failure of digestive organ growth in zebrafish with elevated nucleolar stress

**DOI:** 10.1016/j.isci.2022.104957

**Published:** 2022-08-17

**Authors:** Jinmin Ma, Xuelian Shao, Fang Geng, Shuzhang Liang, Chunxiao Yu, Ruilin Zhang

**Affiliations:** 1 School of Life Sciences, Fudan University, Shanghai 200438, China; 2 TaiKang Medical School (School of Basic Medical Sciences), Wuhan University, Wuhan 430071, China; 3 Hubei Provincial Key Laboratory of Developmentally Originated Disease, Wuhan 430071, China

**Keywords:** Animal physiology, biological sciences, cell biology, molecular biology

## Abstract

Mutations in ERCC2/XPD helicase, an important component of the TFIIH complex, cause distinct human genetic disorders which exhibit various pathological features. However, the molecular mechanisms underlying many symptoms remain elusive. Here, we have shown that Ercc2/Xpd deficiency in zebrafish resulted in hypoplastic digestive organs with normal bud initiation but later failed to grow. The proliferation of intestinal endothelial cells was impaired in *ercc2/xpd* mutants, and mitochondrial abnormalities, autophagy, and inflammation were highly induced. Further studies revealed that these abnormalities were associated with the perturbation of rRNA synthesis and nucleolar stress in a p53-independent manner. As TFIIH has only been implicated in RNA polymerase I-dependent transcription *in vitro*, our results provide the first evidence for the connection between Ercc2/Xpd and rRNA synthesis in an animal model that recapitulates certain key characteristics of ERCC2/XPD*-*related human genetic disorders, and will greatly advance our understanding of the molecular pathogenesis of these diseases.

## Highlights

• Ercc2/Xpd deficiency results in failure of digestive organ growth in zebrafish
• Ercc2/Xpd-deficient intestinal endothelial cells exhibit impaired proliferation
• Mitochondrial abnormalities, autophagy, and inflammation are highly induced
• rRNA synthesis perturbation leads to nucleolar stress in a p53-independent manner

## Graphical abstract

**Figure:**
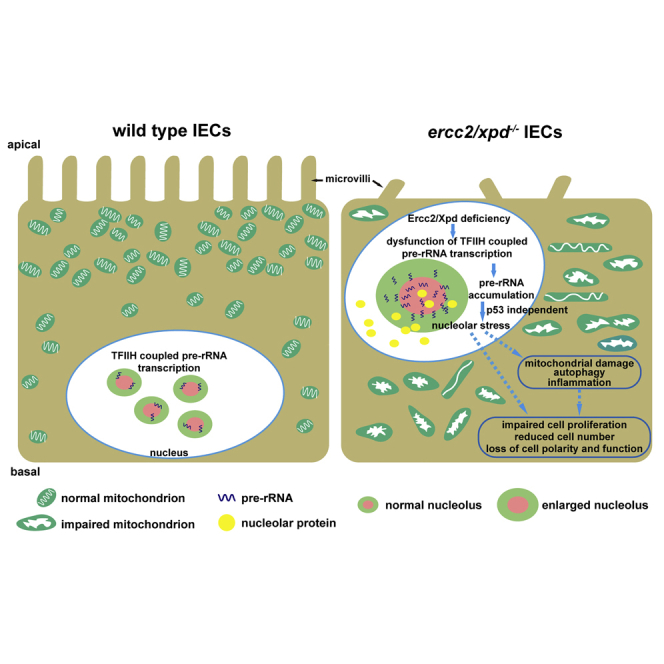

## Introduction

Transcription factor IIH (TFIIH) plays important role in multiple biological processes, including RNA polymerase II-dependent transcription, cell cycle regulation, and DNA repair (Compe and Egly, 2016; Rimel and Taatjes, 2018). This large complex consists of two sub-complexes that are composed of 10 highly conserved subunits in different species (Greber et al., 2017). ERCC2/XPD is a key component of TFIIH because it bridges the two sub-complexes together tightly (Chen et al., 2003; Chen and Suter, 2003), and its helicase activity is important for the opening of DNA duplexes around DNA damage sites during nucleotide excision repair (NER) (Coin et al., 2007; Oksenych and Coin, 2010).

Mutations in *ERCC2/XPD* have been linked to multiple human genetic disorders, including xeroderma pigmentosum (XP), Cockayne syndrome (CS), and trichothiodystrophy (TTD) (Laugel, 2013; Taylor et al., 1997; Ueda et al., 2009). Hundreds of *ERCC2/XPD* mutations have been reported, most of which are missense point mutations located in the helicase domains and result in disrupted NER function and subsequent genomic instability (Cameroni et al., 2010; Fan et al., 2008). Therefore, these diseases were initially defined as DNA repair disorders, and NER deficiency was regarded as the primary cause of most pathological changes. However, the pathological features of these syndromes differ significantly, and the pathogenesis of many clinical symptoms, such as developmental retardation, skeletal abnormalities, hypoplasia of adipose tissue, beta-thalassemia, and premature aging (Lehmann, 2001, 2003; Viprakasit et al., 2001), still remains elusive. Somatic cells in some patients with TTD possess normal NER function (Faghri et al., 2008; Lehmann et al., 1988), but patients with CS and TTD do not exhibit cancer susceptibility, the direct outcome of genomic instability, similar to patients with XP (de Boer et al., 1999; Theil et al., 2014). Patients bearing a NER-null mutation of *XPA*, another disease-causative gene for XP, do not exhibit certain clinical symptoms, such as premature aging (Sugitani et al., 2016). Taken together, growing evidence suggests that disease-causative mutations impair other functions of ERCC2/XPD, and the clinical complexity of these syndromes cannot be explained on the basis of mere DNA repair defects.

To further examine the functions of ERCC2/XPD and the pathogenesis of related diseases, we generated *ercc2/xpd* mutant zebrafish and demonstrated that Ercc2/Xpd played a crucial role in the normal development of zebrafish larvae. Ercc2/Xpd deficiency resulted in failure of digestive organ growth and other abnormalities. Intestinal endothelial cells (IECs) in *ercc2/xpd* mutant larvae exhibited impaired proliferation and loss of cell polarity and function. Mitochondrial abnormalities, autophagy, and inflammation were highly induced as well. Further studies suggested that the perturbation of rRNA synthesis, which then led to nucleolar stress response (NSR), may be responsible for the abnormalities of *ercc2/xpd* mutants in a p53-independent manner. Overall, our data provide the first evidence for the connection between Ercc2/Xpd and rRNA synthesis in an animal model and will shed more light on the molecular pathogenesis of ERCC2/XPD-related human diseases.

## Results

### Ercc2/Xpd is crucial for zebrafish larvae development

Zebrafish *ercc2/xpd* encodes a polypeptide of 760 amino acids that share a highly conserved sequence and functional domains with its human homolog (Figure S1A). Many mutations responsible for ERCC2/XPD-related human diseases are located in the helicase domains near the C-terminus. Therefore, we generated Ercc2/Xpd-deficient zebrafish by targeting the last few exons using the CRISPR/Cas9 knockout system (Figure S1B). A 4-nucleotide (nt) deletion mutation was identified and predicted to cause premature termination of Ercc2/Xpd protein translation (Figures S1B and S1C). Although the transcript in mutant larvae was present at a comparable level to siblings, the protein level was significantly reduced in homozygous mutant larvae (Figure S1D).

**Figure 1:**
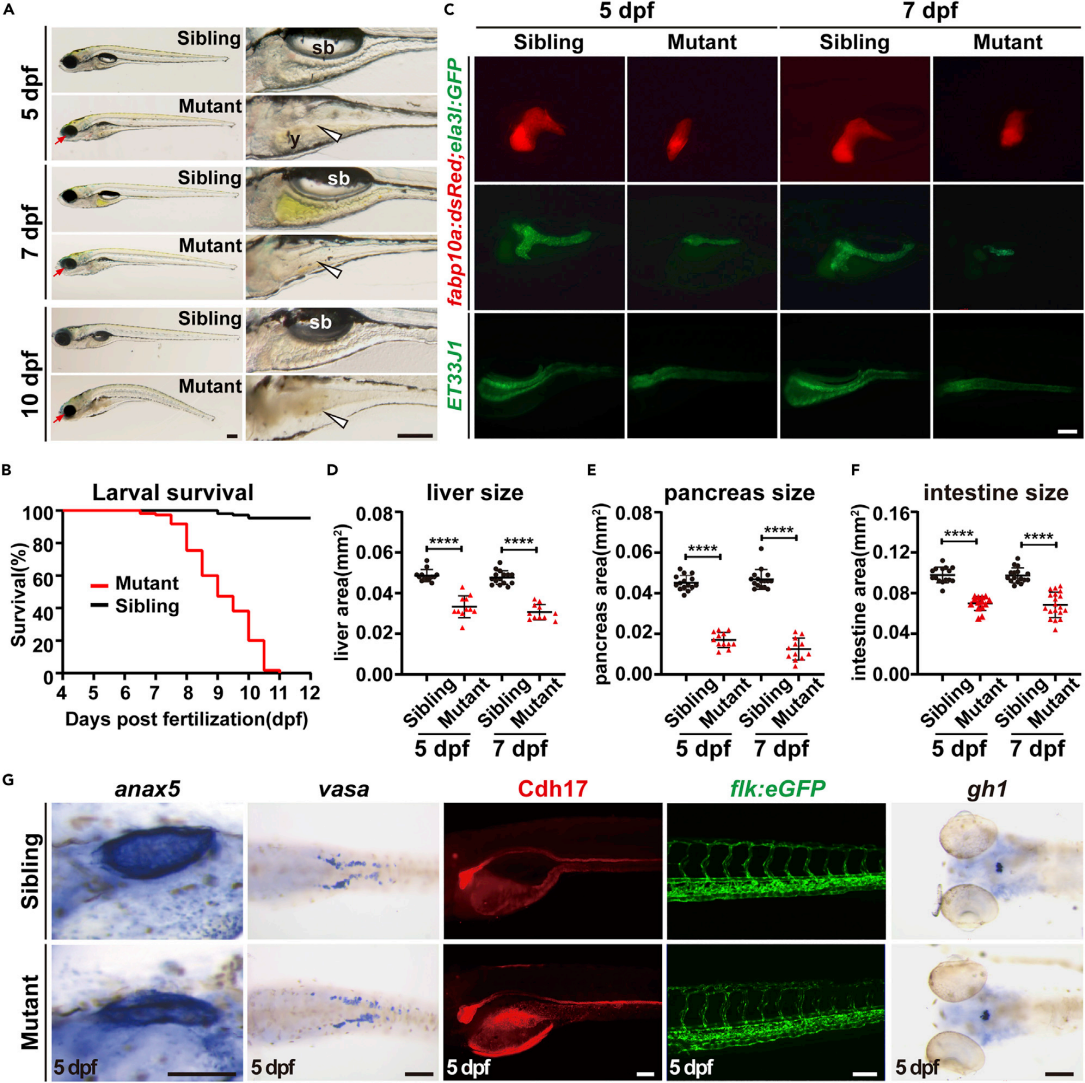
Ercc2/Xpd is crucial for zebrafish larvae development (A) Representative images of *ercc2/xpd* mutants and siblings at the indicated stages. Magnified images of digestive organs are shown on the right. Red arrows indicate microphthalmia in mutants. White arrowheads point to collapse the intestine in mutants. sb, swimming bladder; y, yolk. Scale bars, 200 μm. (B) Survival curves of *ercc2/xpd* mutants and siblings. n = 100 larvae each. (C) Fluorescence images of *Tg(fabp10a:dsRed; ela3l:GFP)* and *ET33J1* fish showing endodermal organs of *ercc2/xpd* mutants and siblings at 5 and 7 dpf. Lateral view, anterior to the left. Scale bar, 100 μm. (D–F) Quantification of liver, pancreas, intestine tube area in *ercc2/xpd* mutants and siblings at 5 and 7 dpf. n ≥ 12. Data are presented as mean ± SD, Student’s *t* test, ∗∗∗∗, p < 0.0001. (G) Whole-mount *in situ* hybridization and fluorescence images showing mesoderm- and ectoderm-derived organs of *ercc2/xpd* mutants and siblings at 5 dpf. Scale bars, 100 μm. See also Figures S1 and S2.

Homozygous *ercc2/xpd* mutant embryos were indistinguishable in gross morphology from their wild-type and heterozygous siblings before 3 days post-fertilization (dpf). Uninflated swim bladder, impaired yolk absorption, and abnormal digestive organ morphology were first observed in mutant larvae at 4-5 dpf and became more severe over time. Other abnormalities, including smaller eyes and jaw malformation, were also observed in the mutants (Figure 1A). By 7-10 dpf, the mutants showed curved bodies, collapsed gut tubes, and gradually lost their viability (Figures 1A and 1B). No homozygous mutants survived to adulthood, but heterozygotes grew normally and were fertile.

Endogenous *ercc2/xpd* transcripts were maternally deposited and widely presented from the 2-cell to 50% epiboly stages but later restricted to the brain and primitive gut. By 3 dpf, the expression of *ercc2/xpd* was enriched in digestive organs (Figure S2). To directly illuminate the endodermal organ abnormalities in *ercc2/xpd* mutants, we bred mutants with the transgenic line *Tg(fabp10a:dsRed; ela3l:GFP)*, which labels the liver and exocrine pancreas in red and green fluorescence, respectively (Cox et al., 2016). *ercc2/xpd* mutants exhibited hypoplastic liver and exocrine pancreas at 5 and 7 dpf (Figure 1C). Quantification of fluorescence areas revealed that mutant livers were reduced to 68% of sibling liver size (0.033 ± 0.005 vs. 0.049 ± 0.003 mm^2^) at 5 dpf and 66% (0.031 ± 0.004 vs. 0.047 ± 0.003 mm^2^) at 7 dpf (Figure 1D). The reduction in exocrine pancreas was more dramatic, with only 38% of sibling pancreas size (0.017 ± 0.004 vs. 0.045 ± 0.004 mm^2^) at 5 dpf and 25% (0.012 ± 0.005 vs. 0.047 ± 0.005 mm^2^) at 7 dpf (Figure 1E). We also assessed intestine development using the enhancer trap line *ET33J1*, which illuminated the gut tube with green fluorescence (Gao et al., 2019; Kondrychyn et al., 2009). The intestine of *ercc2/xpd* mutants exhibited serious defects, which were characterized by a smaller size (0.070 ± 0.007 and 0.068 ± 0.012 mm^2^, 72 and 70% of sibling gut size at 5 and 7 dpf, respectively) and misshapen structure (Figures 1C and 1F).

Because no inflated swim bladders were observed in mutant larvae, we also examined the development of the swim bladder. Whole-mount *in situ* hybridization (WISH) of *anax5*, a gene expressed in swim bladder, showed normal formation of swim bladder in *ercc2/xpd* mutants at 5 dpf (Figure 1G). We further investigated whether Ercc2/Xpd deficiency caused defects in other mesoderm- and ectoderm-derived organs. WISH and immunostaining indicated that mesoderm-derived primordial germ cells (PGCs, examined with *vasa* probe), pronephric duct (examined with Cdh17 antibody), and blood vessels (visualized by *flk:egfp* reporter), as well as ectoderm-derived adenohypophysis (examined with *gh1* probe), were intact in the mutant larvae (Figure 1G). Overall, these data affirmed that Ercc2/Xpd played a crucial role in zebrafish larvae development and was required for the normal development of digestive organs in zebrafish.

### Ercc2/Xpd deficiency results in failure of digestive organ growth

**Figure 2:**
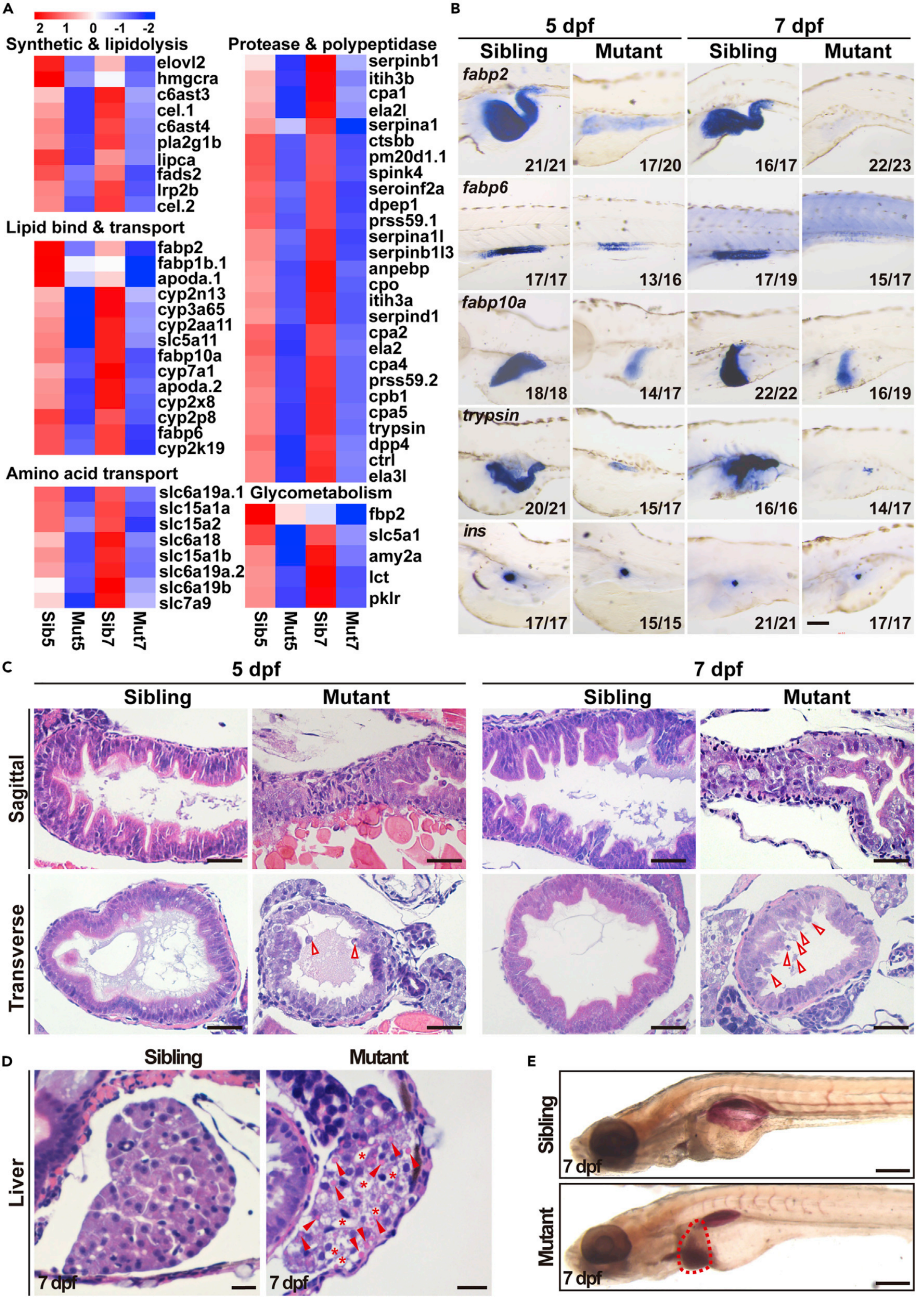
Digestive organ development is defective in *ercc2/xpd* mutants (A) Transcriptomic analysis revealed reduced expression of genes related to digestion and metabolism in *ercc2/xpd* mutants compared to siblings at 5 and 7 dpf. (B) Whole-mount *in situ* hybridization showing reduced expression of marker genes of intestine, liver, and exocrine, but not endocrine, pancreas, in *ercc2/xpd* mutants compared to siblings at 5 and 7 dpf. Lateral view, anterior to the left. Scale bar, 100 μm. See also Figure S3. (C) HE staining of sagittal and transverse sections of *ercc2/xpd* mutants and siblings at 5 and 7 dpf. The intestinal cavity in mutants is smaller or collapsed. Red arrowheads indicate detached cells and cellular debris. Scale bars, 25 μm. See also Figure S4A. (D) HE staining showing vacuolar liver (asterisks) and lipid droplets (arrowheads) in *ercc2/xpd* mutant liver sections at 7 dpf. Scale bars, 25 μm. (E) Whole-mount Oil Red O (ORO) staining showing fatty liver (dashed area) in *ercc2/xpd* mutants at 7 dpf. Lateral view, anterior to the left. Scale bar, 200 μm. See also Figure S4.

To reveal the molecular changes caused by Ercc2/Xpd deficiency, we performed the transcriptomic analysis of sibling and mutant larvae at 5 and 7 dpf. Analysis of differentially expressed genes revealed a dramatically reduced expression of genes encoding peptidases, proteases, and other proteins involved in lipid and amino acid transport as well as in lipo- and glycometabolism in the *ercc2/xpd* mutants (Figure 2A). Most of these genes are digestive tissue specific. To further confirm these results, we performed WISH using a panel of probes. Consistent with the RNA-Seq data, the expression levels of intestinal bulb-specific genes *fatty acid-binding protein 2, intestinal* (*fabp2*), *solute carrier family 15 member 1b* (*slc15a1b*, also known as *pept1*), mid–intestine-specific genes *fatty acid-binding protein 2, ileal* (*fabp6*), *villin1* (*vil1*) and *caudal type homeobox 1b* (*cdx1b*), were sharply down-regulated at 5 dpf and almost undetectable at 7 dpf in the mutant larvae (Figures 2B and S3A). The expression of the liver-specific genes *fatty acid-binding protein 10a* (*fabp10a*), *transferrin-a* (*tfa*), *selenoprotein P2* (*sepp1b*) and *ceruloplasmin* (*cp*), and exocrine pancreas-specific genes *serine protease 1* (*prss1*, also known as *trypsin*), *carboxypeptidase A5* (*cpa5*), *elastase 3 like* (*ela3l*) and *elastase 2 like* (*ela2l*) showed the same trends (Figures 2B, S3B, and S3C). Notably, no apparent defects in the pancreatic islets were observed. Similar expression levels of islet-specific genes, including *preproinsulin* (*ins*, β cell), *glucagon a* (*gcga*, α cell), and *somatostatin 2* (*sst2*, δ cell), were detected in *ercc2/xpd* mutants and siblings (Figures 2B and S3D).

Hematoxylin-eosin (HE) staining was performed to further investigate histological changes in *ercc2/xpd* mutants. In contrast to siblings, which had large intestinal cavities and elaborative epithelium folds, mutants showed collapsed digestive tracts with a thin and unfolded intestinal epithelium at 5 and 7 dpf (Figures 2C and S4A). The intestinal bulbs in mutants were markedly smaller than that in siblings. Detached cells and cellular debris were occasionally seen in the intestinal lumen, and some of the intestinal lumens were almost blocked (Figures 2C and S4A). Analysis of ultrastructure using transmission electron microscopy (TEM) revealed that the intestinal epithelium of siblings was regularly folded and IECs had elaborate apical brush microvilli that were shorter, chaotic and relatively sparse in mutants (Figure S4B). Obvious lipid droplets were observed in mutant livers using HE staining (Figure 2D) and stronger Oil Red O staining also indicated severe hepatocyte steatosis in *ercc2/xpd* mutants (Figure 2E).

**Figure 3:**
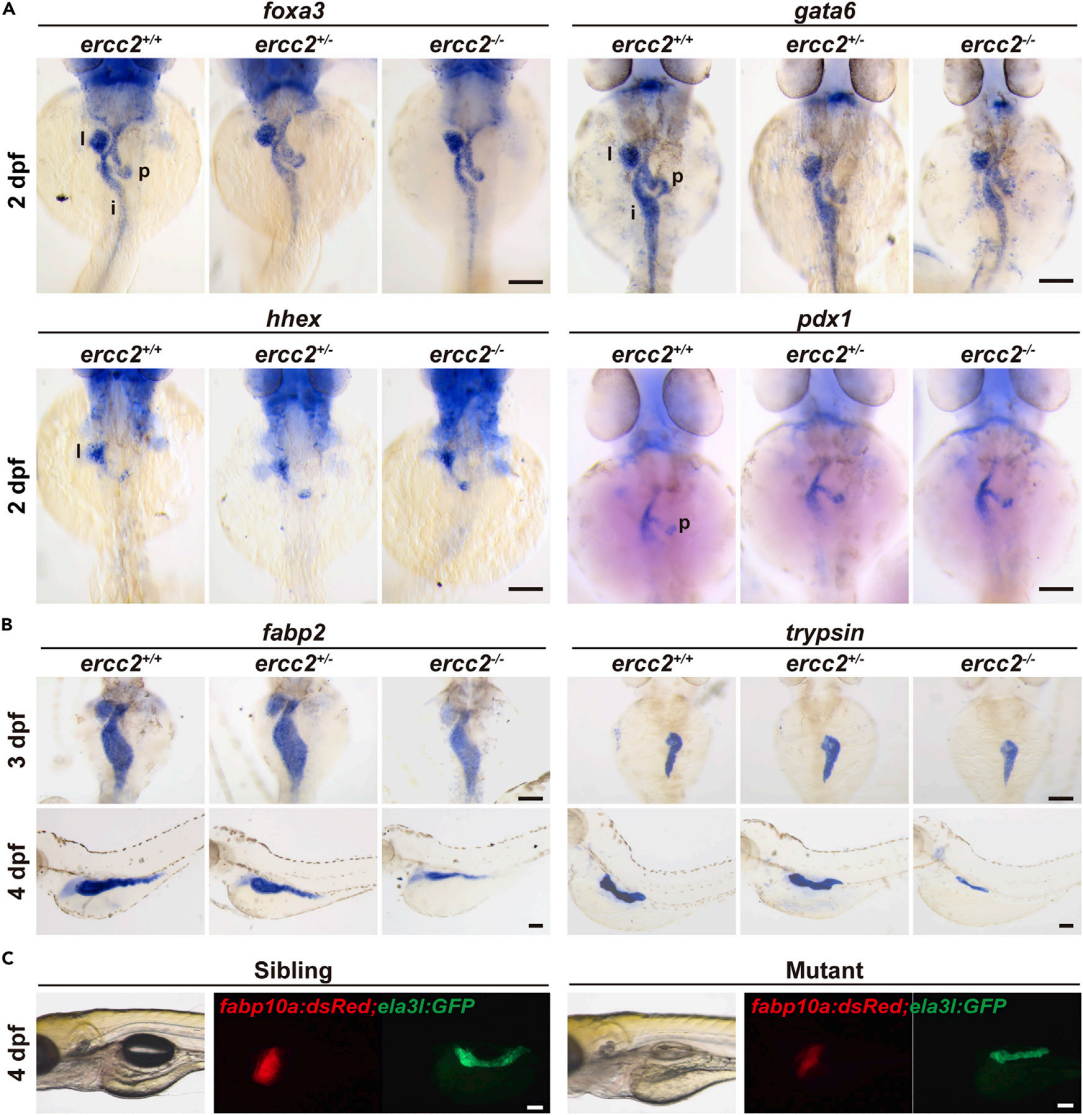
Digestive organ bud initiation is unaffected in *ercc2/xpd* mutants (A) Whole-mount *in situ* hybridization (WISH) of marker genes for digestive organ budding in *ercc2/xpd* mutants and siblings at 2 dpf. Dorsal view, anterior to the top. l, liver; p, pancreas; i, intestine. After individual imaging, embryos were subjected to individual genotyping. Scale bars, 100 μm. (B) WISH of marker genes for early development of digestive organs in *ercc2/xpd* mutants and siblings at 3 and 4 dpf. Upper: dorsal view, anterior to the top; lower: lateral view, anterior to the left. After individual imaging, embryos were subjected to individual genotyping. Scale bars, 100 μm. (C) *Tg(fabp10a:dsRed; ela3l:GFP)* revealed early development of digestive organs in *ercc2/xpd* mutants and siblings at 4 dpf. After individual imaging, embryos were subjected to individual genotyping. Scale bars, 100 μm.

To examine whether bud initiation in digestive organs was affected in the mutants, the early markers including *foxa3*, *gata6* (endoderm cells), *hhex* (hepatoblast) and *pdx1* (precursor cell of the endocrine pancreas) were used in WISH to assess the status of bud formation. After individual imaging, embryos were subjected to individual genotyping (Figure S1E) because the mutants were visually indistinguishable from their siblings at early stages. The initiation of all digestive organ buds in *ercc2/xpd* homozygous mutants was similar to wild-type and heterozygous embryos at 2 dpf (Figure 3A), which indicated that the digestive organ defects were not caused by an errant early differentiation. We further explored the onset of digestive organ defects in *ercc2/xpd* mutants using WISH of *fabp2* and *trypsin* to mark intestine and pancreas development, respectively. Their expression in mutants showed subtle aberrations as early as 3 dpf and was notably down-regulated at 4 dpf (Figure 3B). The *Tg(fabp10a:dsRed; ela3l:GFP)* reporter line also showed a smaller exocrine pancreas and liver in mutants at 4 dpf (Figure 3C). Taken together, these results suggested that the compromised expression of digestive organ genes was not due to a failure of cell fate specification or early developmental defects, but a failure of digestive organ growth in *ercc2/xpd* mutants.

### Ercc2/Xpd-deficient intestinal endothelial cells exhibit loss of cell polarity and impaired proliferation

**Figure 4:**
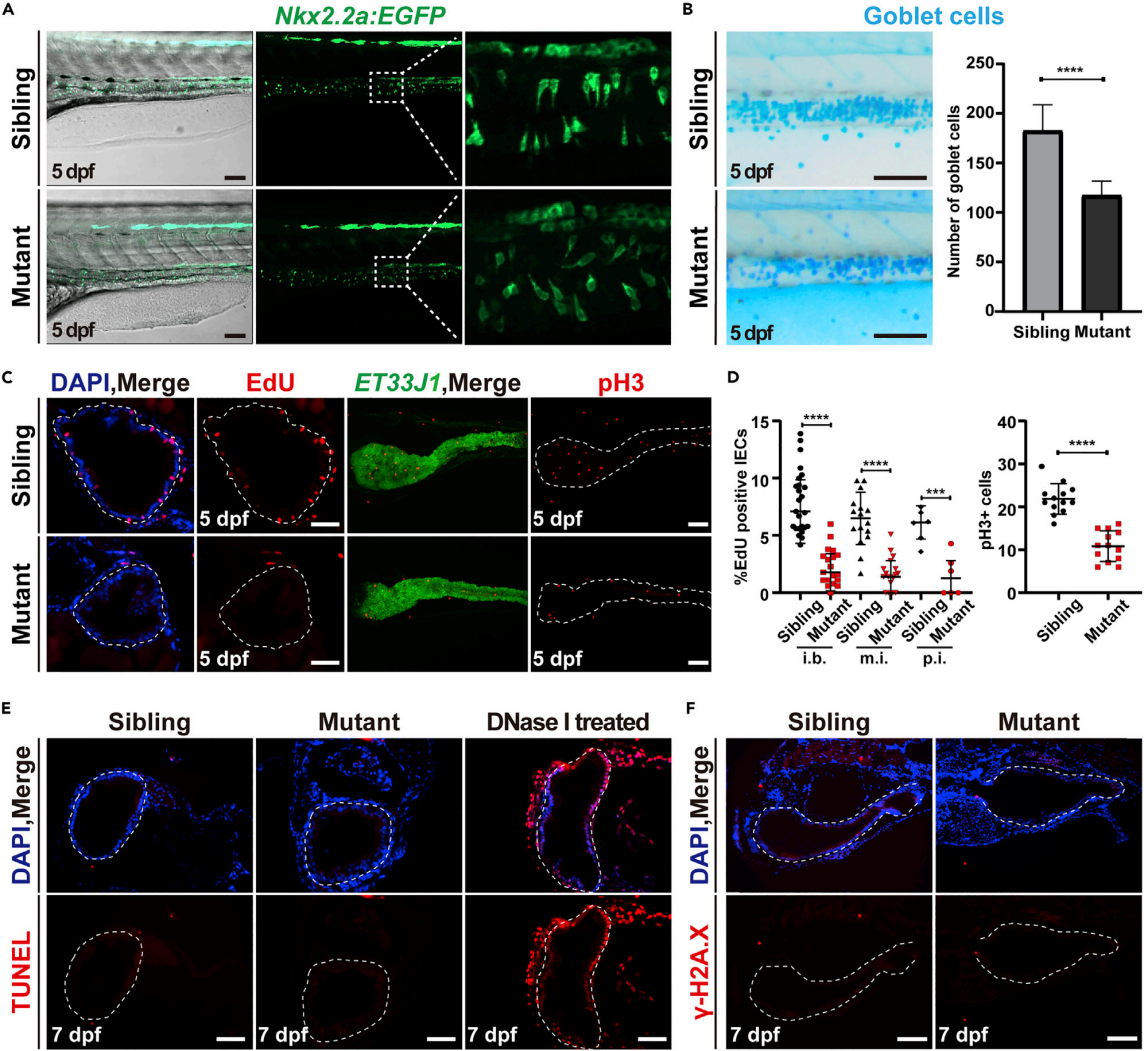
Ercc2/Xpd-deficient intestinal endothelial cells exhibit loss of cell polarity and impaired proliferation (A) *Tg(Nkx2.2a:EGFP)* marked intestinal endocrine cells in *ercc2/xpd* mutants and siblings at 5 dpf. Scale bars, 100 μm. (B) Alcian blue staining showed intestinal goblet cells (left) and quantification of their numbers (right) in *ercc2/xpd* mutants and siblings at 5 dpf. Scale bars, 100 μm. Data are presented as mean ± SD. Student’s *t* test, ∗∗∗∗, p < 0.0001. (C) EdU incorporation assay and immunostaining of the mitotic marker phospho-histone H3 revealed reduced the proliferation of intestinal endothelial cells (IECs) in *ercc2/xpd* mutants compared to siblings at 5 dpf. Scale bars, 100 μm. (D) Quantification of percentages of EdU-positive IECs or pH3-positive cells in different regions of the intestine. i.b., intestinal bulb; m.i., mid-intestine; p.i., posterior intestine. Data are presented as mean ± SD, Student’s *t* test, ∗∗∗∗, p < 0.0001, ∗∗∗, p < 0.001. See also Figure S4D. (E) The TUNEL assay showed no detectable apoptotic signals in IECs in *ercc2/xpd* mutants and siblings compared to the DNase-I-treated positive control at 7 dpf. Scale bars, 100 μm. See also Figure S5A. (F) Immunostaining of γ-H2A.X revealed no increased apoptosis or DNA instability in IECs in *ercc2/xpd* mutants compared to siblings at 7 dpf. Scale bars, 100 μm.

Consistent with the HE staining showing thin and incompact arranged intestinal epithelium, DAPI staining of intestine sections revealed a reduction in IEC nucleus numbers in *ercc2/xpd* mutants (Figures 2C, S4C, and S4D). The IEC nucleus numbers reduced from 213 ± 47, 135 ± 32, and 55 ± 6 per transverse section in siblings to 101 ± 20, 69 ± 19, and 38 ± 5 per transverse section in mutants in the intestinal bulb, mid-intestine, and posterior intestine, respectively, at 5 dpf. The nuclei also lost their basal localization within IECs, as seen in siblings, which indicated a loss of cell polarity (Figures S4B and S4C). The *Tg(Nkx2.2a:GFP)* transgenic line (Pauls et al., 2007) further showed normally differentiated but misoriented intestinal endocrine cells and Alcian blue staining showed reduced numbers of mucin-producing goblet cells (114 ± 14 vs. 183 ± 26) in the intestines of mutants (Figures 4A and 4B). Because of the apparently fewer IECs in mutant larvae, we examined cell proliferation using a 5-ethynyl-2′-deoxyuridine (EdU) incorporation assay and observed a dramatic decrease in the number of EdU-positive IECs in mutants compared to siblings. In general, the percentages of EDU-positive IECs in the intestinal bulb, mid-intestine, and posterior intestine were reduced from 7.1 ± 2.8, 6.5 ± 2.3, and 6.2 ± 1.4 per transverse section in siblings to only 1.8 ± 1.6, 1.4 ± 1.4, and 1.1 ± 1.8 per transverse section in mutants at 5 dpf, respectively (Figures 4C, 4D, and S4D). Consistently, the number of phospho-histone H3 (pH3, a mitotic marker)-positive cells in the whole intestine was also reduced to 50% (11 ± 3.5 vs. 22 ± 3.6) in the mutants compared to the siblings at 5 dpf (Figures 4C and 4D).

We performed a TUNEL assay to assess the apoptosis of IECs in mutants. Notably, no detectable apoptotic signals were observed in intestine or liver sections in mutants or siblings at 7 dpf compared to DNase-I-treated sections, which served as a positive control (Figures 4E and S5A). Acridine orange staining also showed a similar result of no elevated apoptotic signal in mutants at 5 and 7 dpf (Figure S5B). As Ercc2/Xpd plays a vital role in nucleotide excision repair whose disturbance would cause genomic instability, we performed immunostaining of γ-H2A.X, which is a marker of DNA breakage and indicator of the early apoptotic stage (Rogakou et al., 2000). The results indicated no significant increase in γ-H2A.X-positive IECs in *ercc2/xpd* mutants at 7 dpf (Figure 4F). WISH of DNA damage/cell apoptosis-related genes *atr, atm, casp3a,* and *casp3b* revealed comparable expression levels between *ercc2/xpd* mutants and siblings (Figure S5C). We found no elevated senescence-associated β-galactosidase (SA-β-gal) staining or propidium iodide (PI) staining, a widely used dye for pyroptosis detection, in IECs in *ercc2/xpd* mutants, despite strong SA-β-gal staining in the remaining yolks of mutants at 7 dpf (Figures S5D and S5E). Overall, these results indicated that proliferation reduction, but not enhanced cell apoptosis, senescence, or pyroptosis, contributed to the decrease of IECs observed in *ercc2/xpd* mutants.

### Mitochondrial abnormalities, autophagy, and inflammation are highly induced

**Figure 5:**
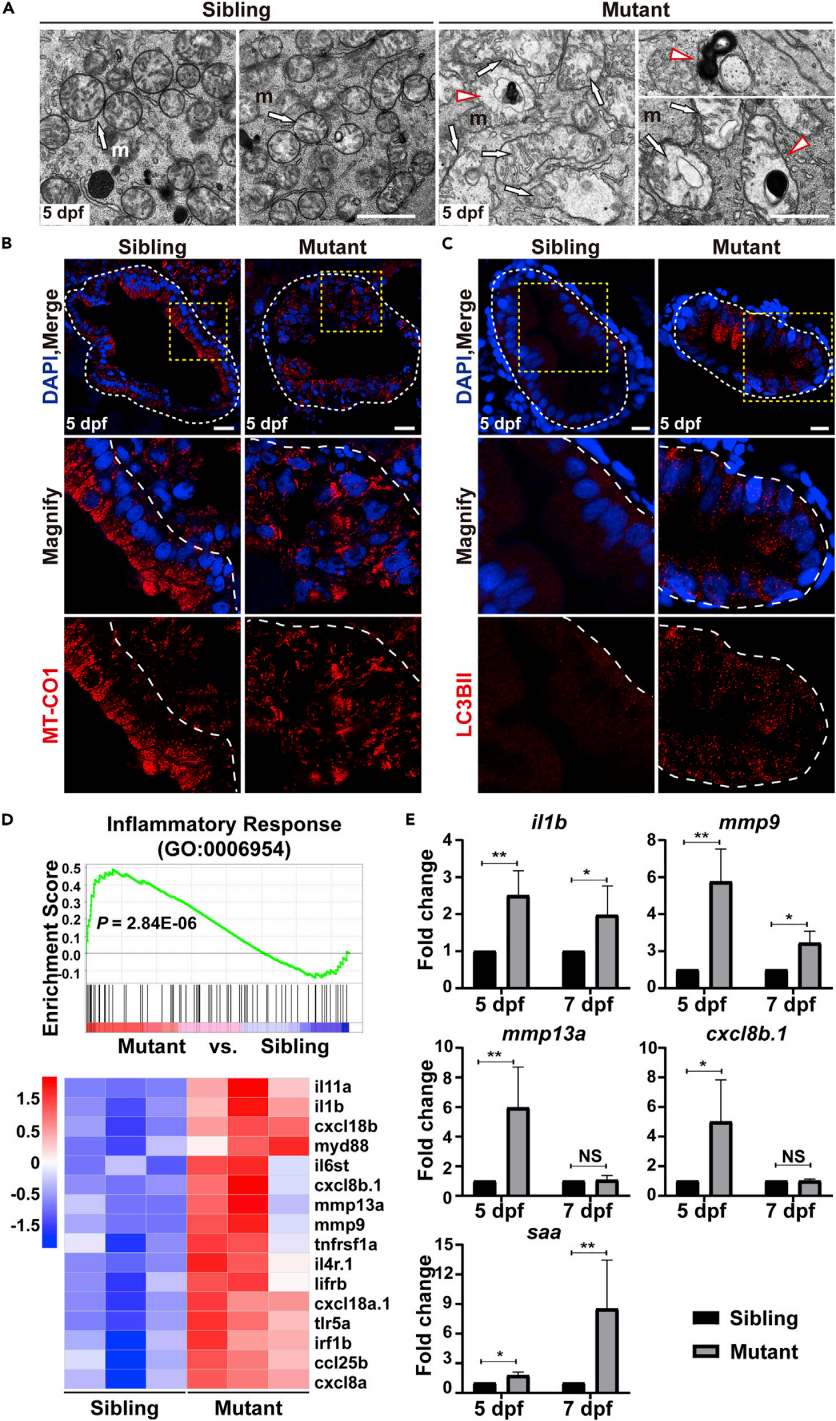
Mitochondrial abnormalities, autophagy, and inflammation are highly induced (A) Transmission electron microscope images of intestinal endothelial cells (IECs) in *ercc2/xpd* mutants and siblings at 5 dpf. Arrows point to swollen and disarrayed mitochondria, red arrowheads point to autophagosome-like vesicles. m, mitochondrion. Scale bars, 1 μm. See also Figures S4B and S6A. (B) Immunostaining of the mitochondrial marker MT-CO1 in IECs in *ercc2/xpd* mutants and siblings at 5 dpf. Areas of dashed boxes are magnified. Scale bars, 20 μm. (C) Immunostaining of the autophagosomal marker LC3BII in IECs in *ercc2/xpd* mutants and siblings at 5 dpf. Areas of dashed boxes are magnified. Scale bars, 10 μm. (D) Transcriptomic analysis revealed increased expression of genes related to inflammation in *ercc2/xpd* mutants compared to siblings at 5 dpf. (E) qPCR verification of inflammation-related gene expression in *ercc2/xpd* mutants and siblings at 5 and 7 dpf. Data are presented as mean ± SD from three independent biological repeats. Student’s *t* test, ∗, p < 0.05, ∗∗, p < 0.01, NS, non-significant. See also Figure S6.

Notably, our TEM images revealed swollen mitochondria with disorganized cristae in Ercc2/Xpd-deficient IECs (Figure 5A, white arrows, Figure S4B) compared to granular mitochondria in sibling IECs. The number of mitochondria in mutant IECs was reduced, and mitochondria seemed more randomly distributed instead of showing apical accumulation as observed in sibling IECs, which is consistent with the results that mutant IECs lost their polarity. Immunostaining of the mitochondrial marker MT-CO1 confirmed these findings. Although abundant mitochondrial signals were present on the apical side of IECs in siblings, the signal was greatly reduced and became sparse in mutant IECs (Figure 5B). These data indicated the existence of mitochondrial dysfunction, which would induce mitophagy to degrade and eliminate damaged mitochondria (Yoo and Jung, 2018). Mitophagy is a common feature in neurodegeneration and aging (Fivenson et al., 2017), which are typical symptoms of ERCC2/XPD-related human diseases.

TEM images also showed many autophagosome-like structures presented in IECs in *ercc2/xpd* mutants but absent in siblings (Figure 5A, red arrowheads, Figure S6A). To determine whether autophagy was induced in IECs in *ercc2/xpd* mutants, we performed immunostaining of LC3B-II, which is a robust marker of autophagosomes (Klionsky et al., 2016), on intestine sections at 5 dpf. The results showed significantly higher numbers of LC3B-II-positive puncta in Ercc2/Xpd-deficient IECs (Figure 5C). To explore the relationship between autophagy and defects in the *ercc2/xpd* mutant, we inhibited autophagy with 50 μM chloroquine (CQ) or 10 mM 3-MA. However, these treatments did not rescue the phenotypes of *ercc2/xpd* mutants but rather slightly enhanced them (Figures S6B and S6C). Therefore, we speculated that autophagy in IECs in *ercc2/xpd* mutant larvae was a self-rescue mechanism when challenged by stress, which is consistent with previous observations in *titania* mutant larvae (Boglev et al., 2013).

Autophagy is generally associated with inflammation. GO enrichment analysis of transcriptomic data revealed that up-regulated genes in *ercc2/xpd* mutants were involved in biological processes related to inflammatory responses. A heatmap of differentially expressed genes indicated that inflammation was highly activated in *ercc2/xpd* mutants at 5 dpf (Figure 5D). Quantitative real-time PCR further confirmed significant up-regulation of the inflammatory-related genes *il1b*, *mmp9,* and *serum amyloid A* (*saa*) at 5 and 7 dpf and *mmp13a*, *cxcl8b* at 5 dpf (Figure 5E). Taken together, these data suggested that inflammation was activated in *ercc2/xpd* mutants and might contribute to the impairment of Ercc2/Xpd-deficient IECs with mitochondrial abnormalities and autophagy.

### Perturbed rRNA synthesis results in nucleolar stress

To determine which stress was responsible for the induction of autophagy in Ercc2/Xpd-deficient IECs, we first detected the expression of several stress-related genes using semi-quantitative RT-PCR, including the redox homeostasis-related genes *nrf2*, *gpx1a*, *prdx4* and *hif1ab* and the endoplasmic-reticulum-stress-related gene *hspa5* (*bip*) (Figures S5G and S5H). The results revealed no significant expression changes between *ercc2/xpd* mutants and siblings. DCFH-DA staining, an indicator of reactive oxygen species (ROS), also exhibited comparable signals in the gut tubes between these two genotypes (Figure S5F).

**Figure 6:**
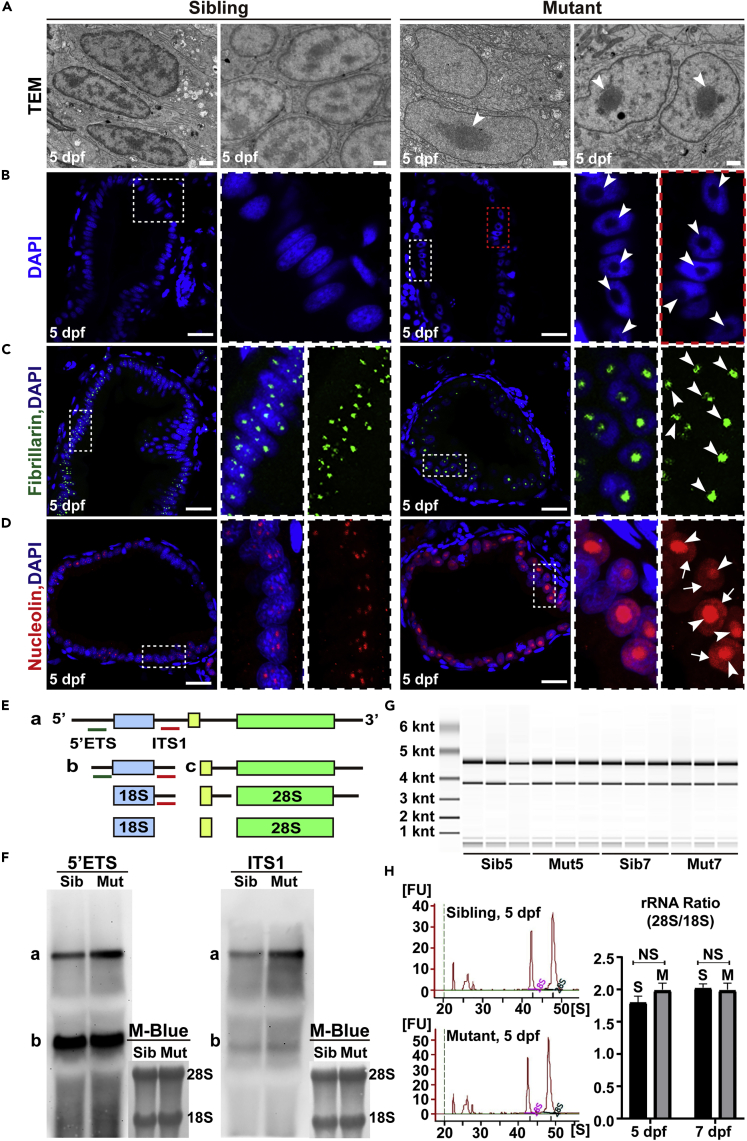
Perturbed rRNA synthesis results in nucleolar stress (A) Transmission electron microscopy images of intestinal endothelial cell (IEC) nuclei in *ercc2/xpd* mutants and siblings at 5 dpf. Arrowheads point to enlarged nucleoli. Scale bars, 1 μm. (B–D) DAPI staining and immunostaining of the nucleolar markers fibrillarin and nucleolin in IECs in *ercc2/xpd* mutants and siblings at 5 dpf. Areas of dashed boxes are magnified. Arrowheads point to enlarged nucleoli, arrows indicate the translocation of nucleolar proteins to the nucleoplasm. Scale bars, 20 μm. See also Figure S7. (E) Schematic diagram showing stepwise processing of the pre-rRNA transcript. a-c corresponds to the rRNA intermediates. 5′ETS and ITS1 probes are indicated with green and red lines, respectively. ETS, external transcribed spacer; ITS, internal transcribed spacer. (F) Northern blot using 5′ETS and ITS1 probes to detect precursor forms of rRNA in *ercc2/xpd* mutants and siblings at 5 dpf. Methylene blue staining was used as a loading control. (G and H) Representative E-Bioanalyzer analysis and measurement of the 28S/18S rRNA ratio in *ercc2/xpd* mutants (M) and siblings (S) at 5 and 7 dpf. Data are presented as mean ± SD, Student’s *t* test, NS, non-significant.

Recent reports showed that ribosome defects triggered autophagy (Boglev et al., 2013; Jia et al., 2015), and TFIIH has been implicated in RNA polymerase I-dependent transcription and rRNA synthesis (Assfalg et al., 2012; Iben et al., 2002; Nonnekens et al., 2013). Our TEM images of IECs showed that *ercc2/xpd* mutant nuclei contained prominently condensed nucleoli compared to more scattered nucleoli in sibling nuclei (Figure 6A). Histological staining revealed enlarged nucleoli devoid of the DAPI signal in Ercc2/Xpd-deficient IECs (Figures 6B and S7A) and hepatocytes, which was also apparent using toluidine blue staining (Figures S7B and S7C). These results suggested the existence of nucleolar stress, which is characterized by the disorganization of nucleolar structure and mislocalization of certain nucleolar proteins (Bennett et al., 2018; Bi et al., 2019; Boulon et al., 2010). To further evaluate the impact of Ercc2/Xpd deficiency on nucleolar structure and functions, we performed immunostaining with different nucleolar markers. The fibrillarin-enriched dense fibrillary component, an important area for early rRNA synthesis and processing in the nucleolus (Raska et al., 1995), was totally disrupted and formed a larger, condensed structure in Ercc2/Xpd-deficient IECs as shown by anti-fibrillarin staining (Figures 6C and S7A). The function of the nucleolus was also perturbed. Nucleolin is an important nucleolar protein involved in rRNA synthesis and processing (Durut and Saez-Vasquez, 2015), and it exhibited altered localization to nucleoplasm in mutant IECs compared to the nucleolar restricted expression in sibling IECs (Figures 6D and S7A).

Defective rRNA synthesis and processing may result in abnormal nucleolar structure and function (Bennett et al., 2018; Zhao et al., 2019). To assess whether Ercc2/Xpd deficiency affected rRNA synthesis, Northern blotting was performed to detect the level of pre-rRNA transcription using 5′ETS and ITS1 probes, and the results showed that the *ercc2/xpd* mutants had a significant accumulation of long pre-rRNA (Figures 6E and 6F, precursor a). The 5′ETS and ITS1 probes showed 2.1- and 3.5-fold higher accumulation of pre-rRNA in *ercc2/xpd* mutants than that in siblings, respectively. E-bioanalyzer analysis of total RNA revealed a comparable rRNA (28S/18S) ratio between mutants and siblings (Figures 6G and 6H), which indicated that the maturation of 18 and 28S rRNA was not affected in *ercc2/xpd* mutant larvae. These results suggested that Ercc2/Xpd deficiency led to a perturbation of rRNA synthesis and subsequent nucleolar stress response, which might induce mitochondrial abnormalities and autophagy and resulted in organ defects in *ercc2/xpd* mutants.

### Ercc2/Xpd deficiency results in hematopoiesis defects

Disruption of rRNA synthesis and subsequent nucleolar stress may lead to severe anemia in humans (Aguissa-Toure et al., 2009; Narla and Ebert, 2010) and zebrafish (Bielczyk-Maczynska et al., 2015; Danilova et al., 2008). Previous studies showed that mutations in ERCC2/XPD resulted in beta-thalassemia in patients with trichothiodystrophy (Tolmie et al., 1994; Viprakasit et al., 2001). Therefore, to investigate whether the nucleolar stress in Ercc2/Xpd-deficient zebrafish mimicked this disease-related phenotype, we examined hematopoiesis in *ercc2/xpd* mutants. The WISH results revealed that embryonic erythrocyte markers *hbae1*, *hbae3,* and *hbbe1* were almost absent in *ercc2/xpd* mutants compared to strong expression in siblings at 5 and 7 dpf (Figure S8A). Similarly, the expression of the T lymphocyte marker *rag1* was dramatically down-regulated at 5 dpf and almost disappeared at 7 dpf in *ercc2/xpd* mutants (Figure S8A). We further examined the timing of this defect and found that primitive hematopoiesis was intact in *ercc2/xpd* mutants, as shown by the expression of *gata1a*, at 1 dpf. The expression of *hbae3* and O-dianisidine staining showed normal erythrocytes at 2 and 3 dpf but slightly reduced numbers at 4 dpf in mutants (Figures S8C and S8D). Tracing the expression of *cmyb* indicated that the onset of hematopoiesis defects in *ercc2/xpd* mutants started as early as 3 dpf because its expression was already significantly decreased at this time point and then was almost undetectable in caudal hematopoietic tissue (CHT) and kidney marrow (KM) at 4 and 5 dpf (Figure S8B). These results suggested that similar to anemia in ERCC2/XPD-related human disorders, hematopoiesis was also disturbed in *ercc2/xpd* mutant zebrafish, which was likely due to nucleolar stress.

### Ercc2/Xpd-deficient phenotypes are independent of an activated p53 response

**Figure 7:**
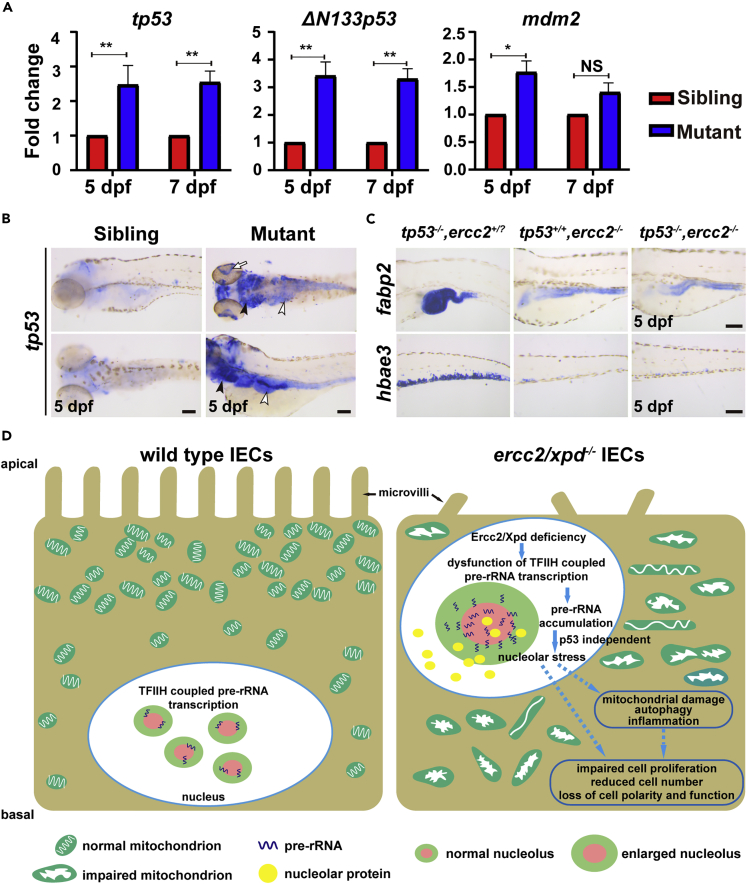
Ercc2/Xpd-deficient phenotypes are independent of an activated p53 response (A) qPCR analysis of expressions of genes associated with *tp53* activation in *ercc2/xpd* mutants and siblings at 5 and 7 dpf. Data are presented as mean ± SD from three independent biological repeats. Student’s *t* test, ∗, p < 0.05, ∗∗, p < 0.01, NS, non-significant. (B) Whole-mount *in situ* hybridization showed a large up-regulation of *tp53* in digestive organs (white arrowheads), retina (white arrow) and arches (black arrowheads) of *ercc2/xpd* mutants at 5 dpf. Scale bars, 100 μm. (C) Whole-mount *in situ* hybridization revealed that *tp53* mutation did not affect *ercc2/xpd* mutant phenotypes in digestive organs or hematopoiesis. Scale bars, 100 μm. (D) Schematic diagram showing the possible mechanism of how Ercc2/Xpd deficiency leads to failure of digestive organ growth. See also Figure S9.

Defects in rRNA synthesis or ribosome biogenesis activate p53 to induce autophagy (James et al., 2014; Maiuri et al., 2010). However, some studies showed that the outcomes of these defects occurred in a p53-independent manner (Jia et al., 2015; Provost et al., 2012). The qRT-PCR analysis revealed markedly increased expression of *tp53* and its target genes *Δ113p53* and *mdm2* in *ercc2/xpd* mutants at 5 and 7 dpf (Figure 7A). WISH also showed a strong accumulation of *tp53* in the digestive organs, eyes, and jaw of *ercc2/xpd* mutants compared to siblings at 5 dpf (Figure 7B). To determine whether p53 signaling contributed to the phenotypes of *ercc2/xpd* mutants, we crossed *ercc2/xpd* mutants into a mutant form of p53 (p53^M214K^) with negligible DNA-binding activity (Berghmans et al., 2005). However, p53 deficiency did not rescue the morphology of *ercc2/xpd* mutants (Figure S9A). WISH revealed similar weak to absent expression of *fabp2* (intestine), *fabp10a* (liver), *trypsin* (exocrine pancreas), and *heae3* (erythrocytes) in *ercc2/xpd* mutants independent of the *tp53* mutant background at 5 dpf, but *tp53* single mutants showed robust expression (Figures 7C and S9B). These data suggested that defects in digestive organs and hematopoiesis were caused in a p53-independent manner in *ercc2/xpd* mutant zebrafish.

## Discussion

### Zebrafish is a valuable animal model for ERCC2/XPD*-*related diseases

Mutations in ERCC2/XPD are linked to multiple human genetic disorders that were initially defined as DNA repair syndromes. However, many clinical features described over the past decade have been hard to elucidate based on DNA repair defects, which suggested that other pathogenic mechanisms caused these diseases. *Ercc2/Xpd* knockout in mice resulted in preimplantation lethality. Other mouse models, including TTD mice bearing the XPD R722W mutation identified in patients with TTD and XPCS mice with the G602D mutation found in patients with XP/CS, partially recapitulated the symptoms of TTD or XP/CS, such as premature aging, brittle hair, cachexia, UV-hypersensitivity and cancer predisposition (Andressoo et al., 2006; de Boer et al., 1998, 2002). Using a TTD mouse model, Compe et al. showed that the dysregulation of peroxisome proliferator-activated receptor (PPAR) target genes may contribute to hypoplasia of adipose tissues (Compe et al., 2005). However, patients with TTD and CS do not have increased skin cancer susceptibility but TTD mice have increased post-UV cancer frequency (de Boer et al., 1999). Therefore, new animal models are needed to further explore the molecular pathogenesis underlying these diseases.

We generated Ercc2/Xpd-deficient zebrafish using CRISPR/Cas9 technology. The morphology of the eyes, jaw, and digestive organs was severely disturbed in *ercc2/xpd* mutant larvae, and the expression of many metabolism-related genes was dramatically down-regulated. Although bud initiation was not affected, digestive organs failed to grow over time. IECs exhibited impaired proliferation and loss of cell polarity. This tissue-specific phenotype may be explained by the distinct expression of *ercc2/xpd,* which was gradually restricted to rapidly proliferating tissues, such as the cerebellum, jaw, retinal epithelium, and primitive gut, at 2 dpf and further enriched in digestive organs at 3 dpf. We further discovered a connection between nucleolar stress and the abnormalities in *ercc2/xpd* mutants (Figure 7D). We confirmed the role of Ercc2/Xpd in the regulation of rRNA synthesis and ribosome biogenesis, which provides a different perspective on ERCC2/XPD*-*related human diseases. Evidence showed reduced levels of other TFIIH subunits in patients with TTD caused by XPD mutations (Dubaele et al., 2003; Vermeulen et al., 2000). However, zebrafish TFIIH has rarely been studied, and no applicable antibody has been reported. Therefore, further studies are needed to examine the levels of other TFIIH subunits in *ercc2/xpd* mutant zebrafish.

### Perturbation of rRNA synthesis contributes to ERCC2/XPD*-*related diseases

Among ERCC2/XPD-related disorders, CS is a rare autosomal recessive disease that presents with multiple organ degeneration and premature aging (Karikkineth et al., 2017; Laugel, 2013). It is caused by mutations in six NER-related proteins: Cockayne syndrome protein A (CSA/ERCC8), Cockayne syndrome protein B (CSB/ERCC6), ERCC2/XPD, XPB, XPF, and XPG. Because patients with CS do not exhibit elevated cancer-prone symptoms like patients with XP (Cleaver et al., 2009), mechanisms other than NER defects may exist. Previous studies demonstrated that CSA/ERCC8 and CSB/ERCC6 were localized in the nucleolus and acted as activators of RNA polymerase I for pre-rRNA transcription (Bradsher et al., 2002; Koch et al., 2014; Lebedev et al., 2008). TFIIE, whose dysfunction also leads to TTD, and TFIIH are required for rRNA synthesis *in vitro*, and perturbation of rRNA synthesis was observed in TTD patient’s somatic cells (Assfalg et al., 2012; Hoogstraten et al., 2002; Iben et al., 2002; Nonnekens et al., 2013; Phan et al., 2021). These studies indicate that ribosomal biogenesis defects may be an important contributor to the clinical symptoms of these diseases. Consistent with this hypothesis, rRNA synthesis was disturbed in *ercc2/xpd* mutants. Severe anemia, e.g., Diamond-Blackfan anemia and Shwachman-Diamond syndrome (Aguissa-Toure et al., 2009; Narla and Ebert, 2010), is a typical feature of human ribosomopathies. Hematopoiesis in *ercc2/xpd* mutants was impaired, and most downstream hematopoietic lineages were dramatically reduced. These phenotypes of *ercc2/xpd* mutants are highly reminiscent of many ribogenesis dysfunction zebrafish mutant lines caused by mutations in *rpl6, sas10*, *rps19*, *nol9,* and *kir1l* (Bielczyk-Maczynska et al., 2015; Jia et al., 2015; Provost et al., 2013; Zhang et al., 2014; Zhao et al., 2019). All of these mutants showed disorders of rRNA processing and perturbation of ribosomal biogenesis, accompanied by impaired development of digestive organs, eyes, and/or hematopoiesis.

TFIIH serves a dual role as a general transcription factor for transcription initiation by eukaryotic RNA polymerase II (Pol II) and as a DNA helicase complex in nucleotide excision DNA repair (NER). Our study found no significant accumulation of DNA damage in the mutant IECs, which suggested that NER dysfunction was not the major cause of the defects. Transcriptomic analysis showed much fewer differentially expressed genes between mutant and sibling groups than that between 5 and 7 dpf within each group, which indicated that the function of RNA polymerase II was not seriously impaired in *ercc2/xpd* mutants either. Taken together, our results provide evidence that the perturbation of rRNA synthesis is an important contributor to the phenotypes of *ercc2/xpd* zebrafish mutants and ERCC2/XPD-related human disease.

### Nucleolar stress induces mitochondrial abnormalities and autophagy

The size of the nucleolus is a valid indicator of the activity of ribosomal synthesis machinery, and nucleolar expansion is used as a hallmark of aging and as a predictive parameter for metabolic health and life expectancy (Correll et al., 2019). We observed prominently enlarged nucleoli in *ercc2/xpd* mutant nuclei using TEM. Immunostaining also revealed the disorganization of the nucleolar structure and mislocalization of certain nucleolar proteins, which indicated the existence of nucleolar stress. Dysfunction of ribosomal biogenesis causes the accumulation of excess free ribosomal proteins, such as RPL5, RPL11, RPL23, RPL26, and RPS14. These free ribosomal proteins bind and inactivate MDM2 and further stabilize p53, which leads to cell-cycle arrest and apoptosis (Lohrum et al., 2003; Zhang et al., 2010). However, p53-independent processes due to ribosome biogenesis defects have also been described, such as in *titania, rpl3, rpl6*, *kri1l,* and *rps19* mutants (Boglev et al., 2013; Jia et al., 2015; Provost et al., 2012; Torihara et al., 2011). An up-regulation of p53 and its target genes was observed in *ercc2/xpd* mutants, but the p53-null allele did not rescue the abnormalities in digestive organs and hematopoiesis.

Autophagy is a crucial stress response pathway to maintain homeostasis or initiate cell death when cells are challenged by various stressors, such as nutrient deprivation, DNA damage, hypoxia, ROS, ER stress, and nucleolar stress (Parzych and Klionsky, 2014). The proliferation of IECs was arrested in the *ercc2/xpd* mutants, while there was no significant increase in cell apoptosis, DNA damage, or pyroptosis. An increased expression of autophagosome markers was observed. However, the inhibition of autophagy did not rescue the phenotypes of *ercc2/xpd* mutants but rather slightly enhanced them, which suggested autophagy might serve as a self-rescue mechanism when challenged by stress as observed in other ribogenesis-defective zebrafish mutants (Boglev et al., 2013). TEM and immunostaining revealed misshapen mitochondria with reduced numbers and mislocalization in the IECs. Mitochondrial dysfunction induces mitophagy to degrade and eliminate damaged mitochondria in many disease settings. Consistent with our observation, previous reports showed that mitochondrial abnormalities were caused by rDNA transcriptional perturbation in CSA- or CSB-deficient cells (Scheibye-Knudsen et al., 2016).

In conclusion, our study suggests that *ercc2/xpd* mutant zebrafish may be a valuable vertebrate model for ERCC2/XPD-related human disorders and ribosomopathies. Further investigations are needed to provide fundamental insights into the molecular pathology of clinical symptoms and explore the specific roles of ERCC2/XPD and ribosome biogenesis in these diseases.

### Limitations of the study

In this study we identify a key connection between Ercc2/Xpd and rRNA synthesis, however, there are several limitations. First, due to the lack of applicable antibodies for other subunits in zebrafish, we cannot unravel if the whole TFIIH complex is unstable in *ercc2/xpd* mutants as in patients with TTD. Second, we have shown that nucleolar stress induces mitochondrial abnormalities and autophagy, yet the underlying molecular mechanisms warrant further investigation. Third, p53 has been reported to respond to nucleolar stress and contribute to the phenotypes in several zebrafish mutants with defective ribosome biogenesis, but is dispensable in some other mutants as in *ercc2/xpd* mutants. Hence, the roles of p53 during nucleolar stress response also require further exploration.

## STAR★Methods

### Key resources table

**Table:** 

REAGENT or RESOURCE	SOURCE	IDENTIFIER
**Antibodies**		

Mouse monoclonal anti-α-Tubulin	Signalway Antibody	Cat#38059
Goat anti-rabbit IgG, HRP conjugated	CWBio	Cat#CW0103S; RRID:AB_2814709
Goat anti-mouse IgG, HRP conjugated	CWBio	Cat#CW0102S; RRID:AB_2736997
Rabbit monoclonal anti-ERCC2/XPD	Abcam	Cat#ab150362
Rabbit polyclonal anti-Nucleolin	Abcam	Cat#ab22758; RRID:AB_776878
Rabbit polyclonal anti-LC3B	Abcam	Cat#ab51520; RRID:AB_881429
Mouse monoclonal anti-Fibrillarin	Abcam	Cat# ab4566; RRID:AB_304523
Rabbit polyclonal anti-γH2A.X (Ser139)	GeneTex	Cat#GTX127342; RRID:AB_2833105
Rabbit polyclonal anti-pH3 (Ser10)	Millipore	Cat#06-570; RRID:AB_310177
Mouse monoclonal anti-MTCO1	Invitrogen	Cat#459600; RRID:AB_2532240
Alexa-Fluor-555 goat anti-rabbit IgG (H+L)	Invitrogen	Cat#A-21428; RRID:AB_2535849
Alexa-Fluor-488 goat anti-mouse IgG (H+L)	Invitrogen	Cat#A-11001; RRID:AB_2534069
Anti-Digoxigenin-AP, Fab fragments	Roche	Cat#11093274910; RRID:AB_2313640

**Chemicals, peptides, and recombinant proteins**		

DIG RNA Labeling Mix	Roche	Cat#11277073910
DIG DNA Labeling Mix	Roche	Cat#11277065910
NBT/BCIP Stock Solution	Roche	Cat#11681451001
Dig Easy Hyb	Roche	Cat#11603558001
Blocking Reagent	Roche	Cat#11096176001
CDP-*Star*	Roche	Cat#12041677001
Nylon Membranes	Millipore	Cat#11417240001
ssRNA ladder	NEB	Cat#N0362S
EnGen® Spy Cas9 NLS	NEB	Cat#M0646
T7 RNA Polymerase	NEB	Cat#M0251
Torula RNA	Sigma-Aldrich	Cat#R5636
Paraformaldehyde	Sigma-Aldrich	Cat#P6148
Formaldehyde solution	Sigma-Aldrich	Cat#F8775
Oil Red O	Sigma-Aldrich	Cat#O0625
O-Dianisidine	Sigma-Aldrich	Cat#D9143
Alcian Blue 8GX	Sigma-Aldrich	Cat#A5268
Proteinase K	Sigma-Aldrich	Cat#P2308
1-phenyl 2-thiourea (PTU)	Sigma-Aldrich	Cat#P7629
MEGAshortscript T7 Transcription Kit	Thermo	Cat#AM1354
SYBR® Green Master Mix	Thermo	Cat#A25742
TRIzol	Thermo	Cat#10296010
FastDigest BamHI	Thermo	Cat#FD0054
RIPA buffer	Beyotime Biotech	Cat#P0013B
Protease inhibitor cocktail (100×)	Beyotime Biotech	Cat#P1005
Chloroquine	Selleck	Cat#S4157
3-MA	Selleck	Cat#S2767
Vectashield Mounting Medium with DAPI	Vector	Cat#H1200

**Critical commercial assays**		

ReverTra Ace® qPCR RT Master Mix with gDNA Remover Kit	TOYOBO	Cat#FSQ-301
MEGAshortscript™ T7 Transcription Kit	Invitrogen	Cat# AM1354
MEGAclear Transcription Clean-Up Kit	Invitrogen	Cat# AM1908
The Click-iT™ EdU Alexa Fluor™ 647 Flow Cytometry Assay Kit	Invitrogen	Cat#C10419
*In Situ* Cell Death Detection Kit	Roche	Cat#12156792910
SigmaSpin Sequencing Reaction Clean-Up Kit	Sigma-Aldrich	Cat#S5059
Hieff Clone® Zero TOPO-Blunt Kit	Yeasen Biotech	Cat#10910ES20
MightyAmp Genotyping Kit	Takara	Cat#R074A
Senescence Cells Histochemical Staining Kit	Sigma-Aldrich	Cat#CS0030
Enhanced BCA Protein Assay Kit	Beyotime Biotech	Cat#P0010
Hematoxylin and Eosin Staining Kit	Beyotime Biotech	Cat#C0105
Reactive Oxygen Species Assay Kit	Beyotime Biotech	Cat#S0033
Toluidine-blue-o staining kit	Solarbio Biotech	Cat#G3663
Clarity™ Western ECL Substrate	Bio-Rad	Cat#1705060

**Deposited data**		

Raw data of RNA-seq	This paper	GEO:GSE184305

**Experimental models: Organisms/strains**		

Zebrafish: *Tg (flk:egfp)*	ZFIN	ZDB-ALT-050916-14
Zebrafish: *Tg(fabp10a:dsRed; ela3l:GFP)*	(Cox et al., 2016)	N/A
Zebrafish: *Et(krt4:EGFP)*^*sqet33J1*^	(Kondrychyn et al., 2009)	N/A
Zebrafish: *Tg(Nkx2.2a:GFP)*	(Pauls et al., 2007)	N/A
Zebrafish: p53^M214K^	(Berghmans et al., 2005)	N/A
Zebrafish: *ercc2/xpd*^*-/-*^	This paper	N/A

**Oligonucleotides**		

Primers for WISH probes, Northern Blot probes, RT-PCR, qRT-PCR and Genotyping	This paper	see Table S1

**Software and algorithms**		

ImageJ	NIH	https://imagej.nih.gov/ij/
GraphPad Prism 9.0	GraphPad	http://www.graphpad.com/scientific-software/prism/
Zen software	Zeiss	http://www.zeiss.com/corporate/en_de/global/home.html
SPSS software	IBM	https://www.ibm.com/cn-zh/analytics/spss-statistics-software

### Resource availability

#### Lead contact

Further information and requests for resources and reagents should be directed to and will be fulfilled by the lead contact, Ruilin Zhang (zhangruilin@whu.edu.cn).

#### Materials availability

Zebrafish line generated in this study will be shared by the lead contact upon request.

### Experimental model and subject details

Zebrafish were raised and maintained under standard conditions. Zebrafish used in this study included wild-type AB strain, mutant line p53^M214K^, transgenic lines *Tg(flk1:eGFP)*, *Tg(Nkx2.2a:GFP)*, *Tg(fabp10a:dsRed;ela:eGFP)* and *Et(krt4:EGFP)*^*sqet33J1*^. Embryos and larvae were staged according to their morphology (Kimmel et al., 1995). To prevent pigmentation, embryos were incubated with 0.003% PTU (1-phenyl-2-thiourea) in E3 water from 24 hpf. All experiments were performed according to institutional and national animal welfare guidelines and were approved by Fudan University Institutional Animal Care and Use Committee (IACUC).

### Method details

#### Generation of *ercc2/xpd* mutant

*ercc2/xpd* mutants were generated using CRISPR/Cas9 genome-editing technique. Single guide RNA (sgRNA) target site was identified via CRISPRscan website (www.crisprscan.org). The sgRNA transcription template was PCR amplified and then transcribed using MEGAshortscript T7 Transcription Kit (Li et al., 2020) and co-injected with 2 μM Cas9 protein in a final concentration of 50 ng/μl into embryos at one-cell stage.

#### Quantitative real-time PCR

Total RNA from whole larvae was isolated at the indicated stages using the TRIzol Reagent following manufacturer’s instruction. cDNA was synthesized with a ReverTra Ace qPCR kit. The gene expression profile was carried out on biological triplicates with PowerUp SYBR Green Mix, and normalized by β-actin as internal control. Sequences of primers used were listed in Table S1.

#### RNA-seq analysis

30 larvae of *ercc2/xpd* mutants or siblings were pooled as one sample and three samples for each group at 5 dpf or 7 dpf were used for subsequent experiments. RNA-Seq analysis was conducted by Novogene Biotechnology and the sequencing data had been uploaded to Gene Expression Omnibus (GEO) database. Sequenced raw reads were mapped to the zebrafish GRCz11 reference genome. The number of mapped reads was normalized and converted to FPKM for quantification of gene expression change.

#### Whole-mount *in situ* hybridization

WISH was performed using Digoxigenin-labeled RNA antisense probes (Li et al., 2020; Wang et al., 2021). Briefly, zebrafish embryos were fixed in 4% paraformaldehyde (PFA) overnight at 4°C. After rinsed with PBS, embryos were digested with 10 μg/ml proteinase K in PBST (0.1% Tween 20 in PBS) and re-fixed with 4% PFA for 20 min, followed by pre-hybridization in hybridization buffer for 3 to 5 h at 68°C, then incubated overnight with probes (1 ng/μl) diluted in hybridization buffer at 68°C. Detection was performed using 1:4000 dilution of anti-Digoxigenin-AP antibody and visualized by NBT/BCIP substrate reaction. To generate antisense riboprobes, transcription templates were amplified from cDNA using specific primers listed in Table S1, and subcloned into pGEM-T Easy vector. Linearized constructs were transcribed *in vitro* using T7 RNA polymerase with DIG RNA Labeling Mix. *gata1*, *rag1* and *cymb* probes were kindly gifted by Dr.Yuanhua Cai. For experiments detecting gene expression before 4 dpf when the mutant phenotypes cannot be visually distinguished, individual genotyping was performed after individual imaging with a Leica M205FA microscope.

#### Immunofluorescence staining

For whole-mount immunofluorescence detection, fixed zebrafish embryos were rinsed with PBSTx (0.3% Triton X-100 in PBS) and blocked in blocking buffer for 2 h at room temperature, then incubated with primary antibody overnight at 4°C and further with secondary antibody for 2 h at room temperature. Images were taken using a Leica M205FA microscope. For immunofluorescence staining performed on sections (Shao et al., 2020), embryos were fixed in 4% PFA for 1.5 h at room temperature and dehydrated in 30% sucrose, then embedded in OCT solution and further sectioned at 10 μm thickness with a Leica cryostat. Cryosections were washed three times in PBST (0.5% Triton X-100, 0.1% Tween 20 in PBS) and incubated in blocking buffer at 37°C for 1 h, then replaced with diluted primary antibody in blocking buffer overnight at 4°C, further incubated with secondary antibody and subsequently mounted with Vectashield Mounting Medium with DAPI for imaging with a Zeiss LSM880 confocal microscope. The primary antibodies used in this study were anti-phospho-histone H3 (Ser10), anti-Cdh17 (gifted by Dr. Ying Cao), anti-γ-H2A.X, anti-LC3B, anti-Fibrillarin and anti-Nucleolin. The secondary antibodies included Alexa-Fluor-555 goat anti-rabbit IgG (H+L) and Alexa-Fluor-488 goat anti-mouse IgG (H + L).

#### EdU labeling

The larvae were incubated with 500 μM EdU for 3 h in E3 water with 2% DMSO at 5 dpf. After rinsed with PBS solution to stop labeling, larvae were fixed in 4% PFA and prepared for cryosections. The CLICK-IT reaction for EdU labeling was performed according to the manufacturer’s instruction.

#### Histochemical staining

Histochemical staining was performed on paraffin-embedded sections according to manufacturer protocols with hematoxylin-eosin staining kit and toluidine-blue-o staining kit. Intestinal goblet cells were stained using Alcian blue. Briefly, embryos were fixed in 4% PFA, rinsed with acid alcohol (70% ethanol and 0.37% HCl in PBS), then stained with 0.1% Alcian blue 8GX in acid alcohol overnight at room temperature and stored in 70% glycerol for imaging. Whole-mount larvae were incubated respectively in 2 μg/mL acridine orange, 10 μg/mL propidium iodide or 10 μM DCFH-DA for 30–60 min at 28.5°C, or in 0.5% oil red/propanediol overnight at room temperature, then followed standard protocols for corresponding histochemical staining. TUNEL assay was performed on cryosections using an *In Situ* Cell Death Detection Kit. To detect cellular senescence, β-galactosidase assay was performed on cryosections using a Senescence Cell Histochemical Staining Kit. The images were taken using a Leica M205FA microscope or an Olympus IX83 microscope.

#### Immunoblotting

Larvae were homogenized in cold RIPA lysis buffer supplemented with complete proteinase inhibitors. Protein lysates were separated on 12% SDS-PAGE and transferred to PVDF membranes according to standard protocol. The membranes were then incubated with anti-ERCC2/XPD, anti-α-tubulin, and further with HRP-anti-rabbit IgG or HRP-anti-mouse IgG secondary antibody. Signals were visualized with Clarity Western ECL Substrate using a ChemiScope series system (Clinx).

#### Northern blot

Total RNA from zebrafish larvae at 5 dpf was separated by electrophoresis on 1.2% agarose gel containing 2% formaldehyde in 1×MOPS and blotted onto a Hybond-N^+^ nylon membrane. RNA blots were cross-linked to the membrane by UV irradiation and subsequently visualized using methylene blue staining. After pre-hybridization in DIG Easy Hyb for 2 h at 50°C, membrane was probed with Digoxigenin-labeled probes in DIG Easy Hyb (25 ng/mL) overnight at 50°C and then blocked in blocking buffer. Detection was performed using 1:10000 dilution of anti-Digoxigenin-AP antibody in blocking buffer and visualized by CDP-*Star* substrate reaction according to the manufacturer’s instructions. Digoxigenin-labelled probes of zebrafish external transcribed spacer (5’ETS) and internal transcribed spacer 1 (ITS1) were generated from PCR amplified products using primers listed in Table S1, with the DIG DNA Labeling Mix.

#### Quantitation of 18S and 28S rRNA levels

Total RNA extracted from *ercc2/xpd* mutant or sibling larvae were analyzed on an Agilent 2100 E-Bioanalyzer according to the manufacturer’s instructions.

#### Small molecule treatment

Embryos were incubated with small molecule chemicals in embryo medium at 28.5°C until collection. Final concentrations of chemicals used in this study were 50 μM chloroquine, 10 mM 3-MA.

#### Transmission electron microscopy

Zebrafish larvae were fixed with 2.5% glutaraldehyde in 0.1 M phosphate buffer for 2 h at room temperature, then washed in PBS and post-fixed in 2% OsO_4_. After dehydrated through an ethanol gradient series and acetone rinses, the samples were embedded in resin for section. Cross sections (80 nm) of intestine were stained with uranyl acetate and lead citrate successively, and imaged with a FEI Tecnai G2 Spirit Twin electron microscope.

### Quantification and statistical analysis

ImageJ software was used to measure the size of intestine, liver, exocrine pancreas and to count the numbers of proliferating cells. Values were presented as mean ± SD. Statistical significance was defined as ∗, p < 0.05, ∗∗, p < 0.01, ∗∗∗, p < 0.001, ∗∗∗∗, p < 0.0001, ns., non-significant, determined by Student’s *t* test using SPSS and Graphpad Prism 9.0 software.

## Data Availability

• RNA-seq data have been deposited at GEO and are publicly available as of the date of publication. Accession numbers are listed in the key resources table. All data reported in this paper will be shared by the lead contact upon request. • No original code was reported in this study. • Any additional information required to reanalyze the data reported in this paper is available from the lead contact upon request.
