# Supplementary material for: Ercc2/Xpd deficiency results in failure of digestive organ growth in zebrafish with elevated nucleolar stress
Source: iScience. 2022 Aug 17;25(9):104957. doi: 10.1016/j.isci.2022.104957 (PMC9440294; doi:10.1016/j.isci.2022.104957)
Supplement: Document S1. Figures S1–S9 and Table S1 [file mmc1.pdf]

iScience, Volume *25*

Supplemental information

Ercc2/Xpd deficiency results in failure of digestive organ growth in zebrafish with elevated nucleolar stress

Jinmin Ma, Xuelian Shao, Fang Geng, Shuzhang Liang, Chunxiao Yu, and Ruilin Zhang

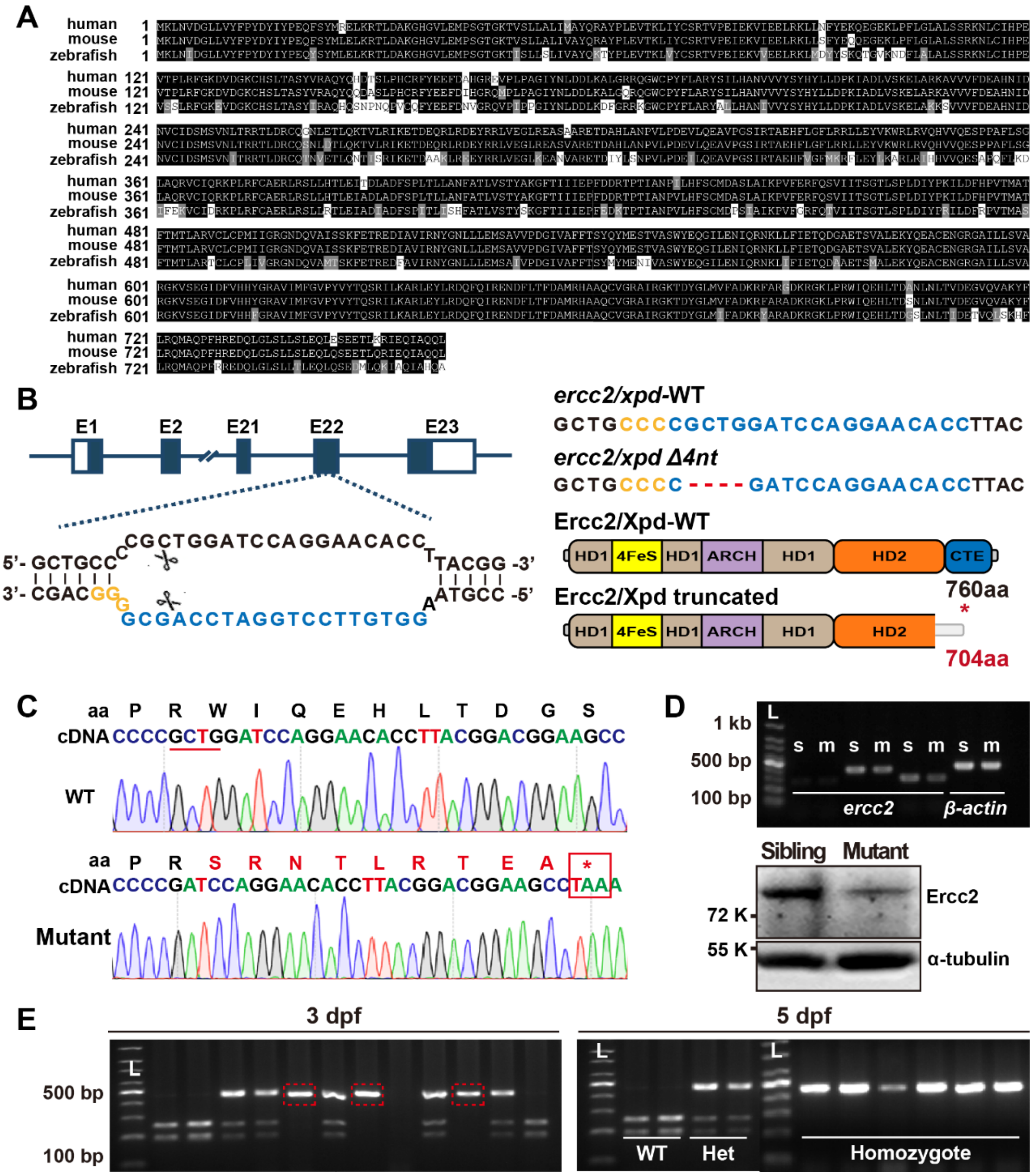

Figure S1. Generation and identification of *ercc2/xpd* mutant zebrafish, related to Figure 1

(A) Sequence alignment of human, mouse and zebrafish ERCC2/XPD proteins. Dark shading indicates identical amino acids; gray shading indicates conserved amino acids.

(B) Left panel: Schematic diagram of zebrafish *ercc2/xpd* genomic locus with sgRNA target site (blue) and PAM sequence (yellow). Right panel: Deletion mutation (red dashes) identified in *ercc2/xpd* gene. The 4-nucleotide deletion is predicted to result in a 704 aa truncated protein. Functional protein domains are annotated.

(C) Sanger sequencing of *ercc2/xpd* cDNA revealed transcripts with a 4-nt deletion (red line) in mutants at 5 dpf, which was predicted to cause frameshift mutation and result in truncated proteins.

(D) Upper: Semi-quantitative RT-PCR analysis showed comparable *ercc2/xpd* transcript levels between mutants (m) and siblings (s) at 5 dpf. Three pairs of primers were used. *β-actin* was used as control. L, DNA ladders. Bottom: Western blot displayed a significant reduction of Ercc2/Xpd protein in

mutants at 5 dpf. α-tubulin was used as loading control.

(E) Embryos and larvae at 2-4 dpf were subjected to individual genotyping with BamHI digestion after individual imaging. Results of *ercc2/xpd* mutants and siblings at 5 dpf were shown as examples. Wild type allele could be cut into two fragments while mutant allele could not. Red dashed boxes indicate homozygous mutants.

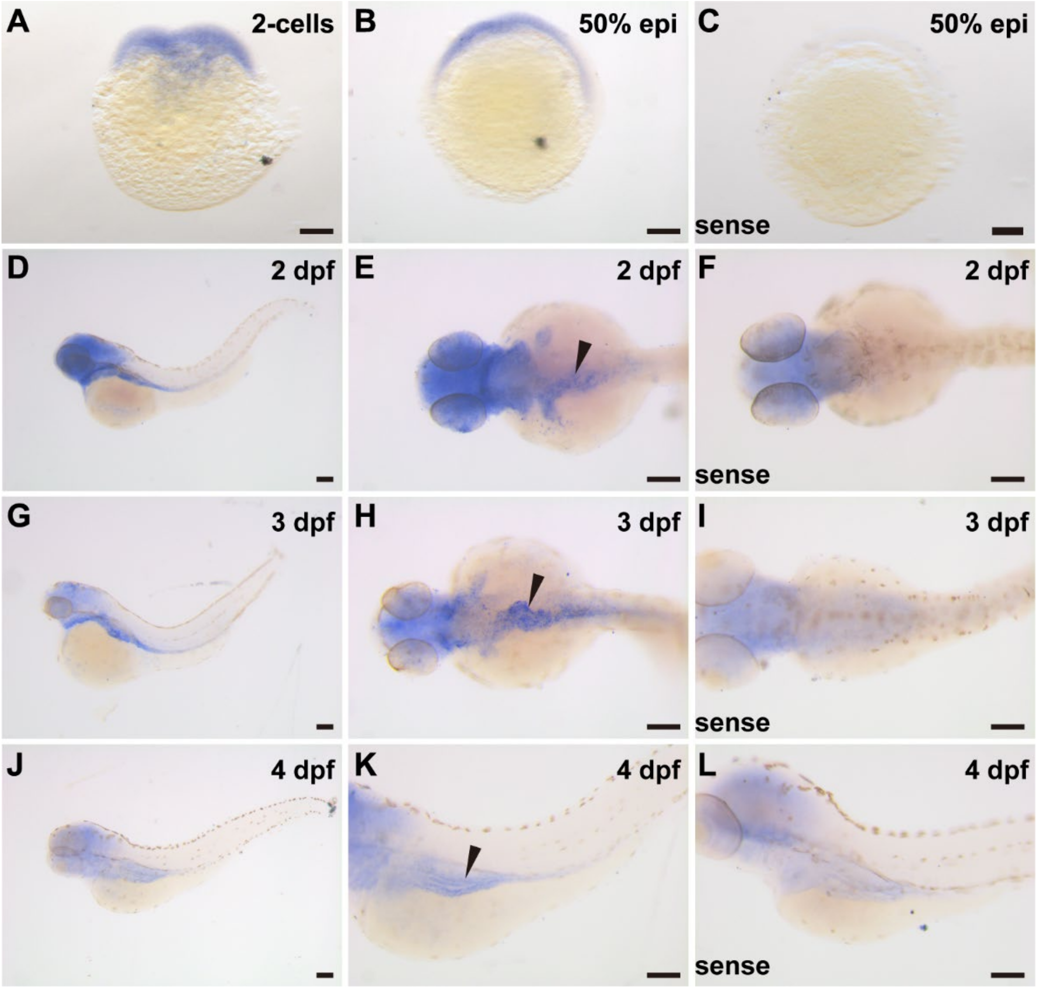

Figure S2. Expression pattern of *ercc2/xpd* during zebrafish early development, related to Figure 1

(A-L) Whole-mount *in situ* hybridization of *ercc2/xpd* at the indicated developmental stages. Arrowheads indicate the intestine tubes. C, F, I, L, sense strand RNA used as control. Scale bars,100 μm.

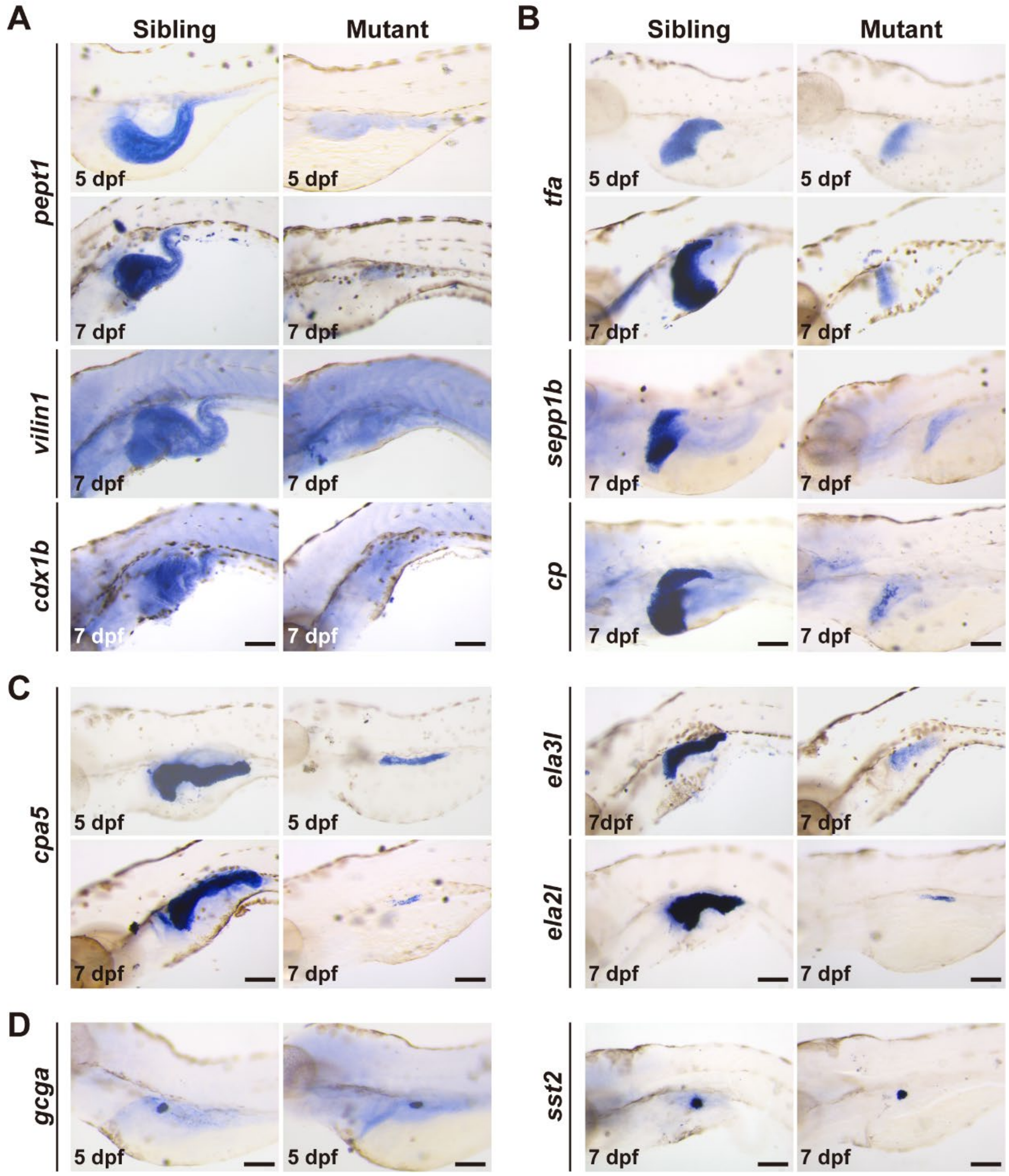

Figure S3. Defective digestive organ development in *ercc2/xpd* mutants, related to Figure 2

(A-D) Whole-mount *in situ* hybridization showed reduced expression of marker genes of intestine (A), liver (B) and exocrine pancreas (C), but not endocrine pancreas (D), in *ercc2/xpd* mutants compared to siblings at the indicated stages. Lateral view, anterior to the left. Scale bars, 100 μm.

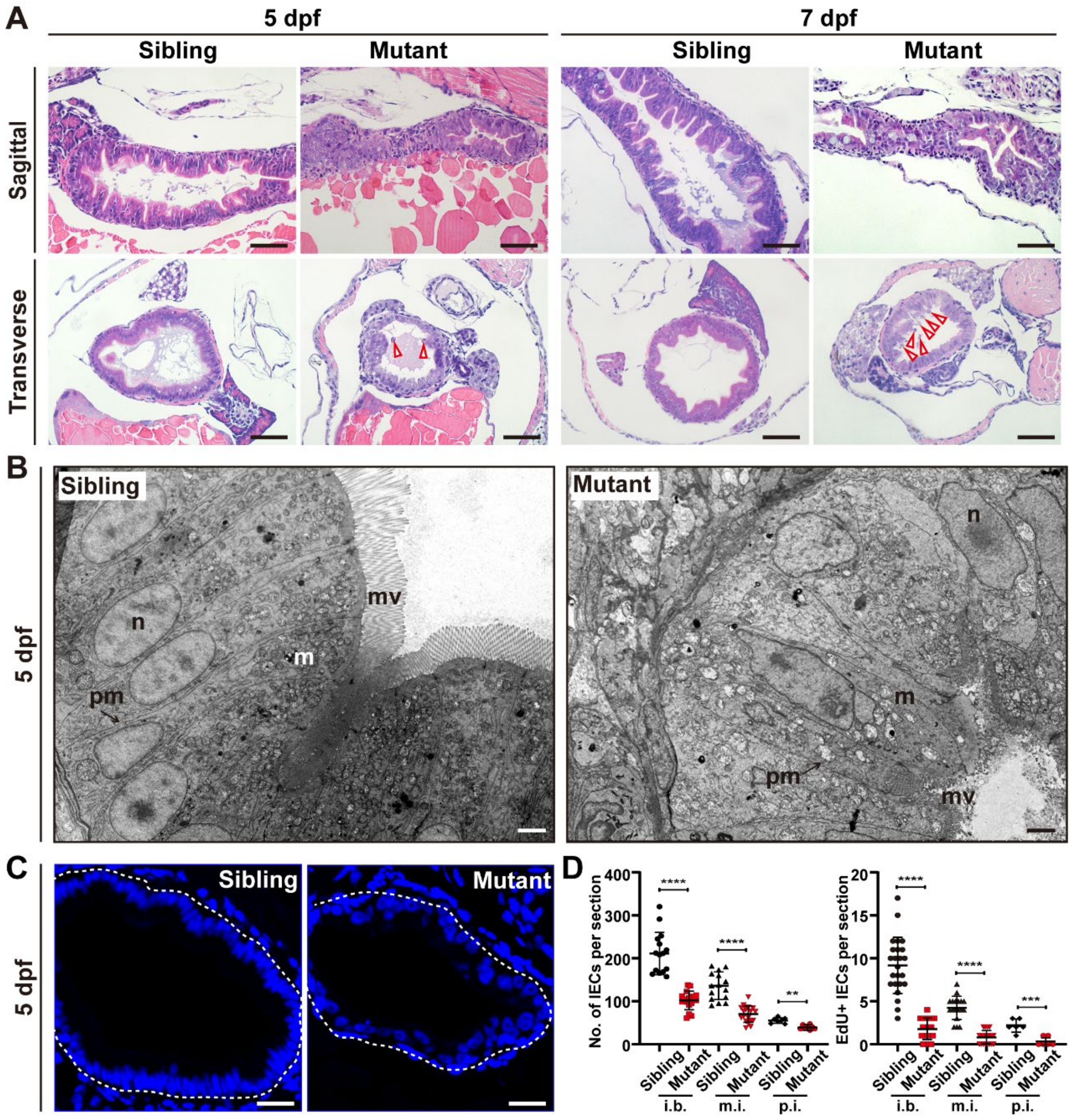

Figure S4. Intestinal defects in *ercc2/xpd* mutants, related to Figure 2, Figure 4 and Figure 5

(A). HE staining of sagittal and transverse sections of *ercc2/xpd* mutants and siblings at 5 and 7 dpf. The intestinal cavity in mutants is smaller or collapsed. Red arrowheads indicate detached cells and cellular debris. Scale bars, 50 μm.

(B). Transmission electron microscopy images of intestinal endothelial cells (IECs) in *ercc2/xpd* mutants and siblings at 5 dpf. Mutant IECs have shorter and relatively sparse microvilli (mv) compared to that in siblings. n, nuclei; pm, plasma membranes. Scale bar, 2 μm.

(C). DAPI staining of intestinal transverse sections in *ercc2/xpd* mutants and siblings at 5 dpf. Dashed lines outline the intestinal epithelium. Scale bars, 20 μm.

(D). Quantification of IEC and EdU-positive IEC numbers in different regions of the intestine. i.b., intestinal bulb; m.i., mid-intestine; p.i., posterior intestine. Data are presented as mean ± SD, Student's t-test, **, $P < 0.01$, ***, $P < 0.001$, ****, $P < 0.0001$.

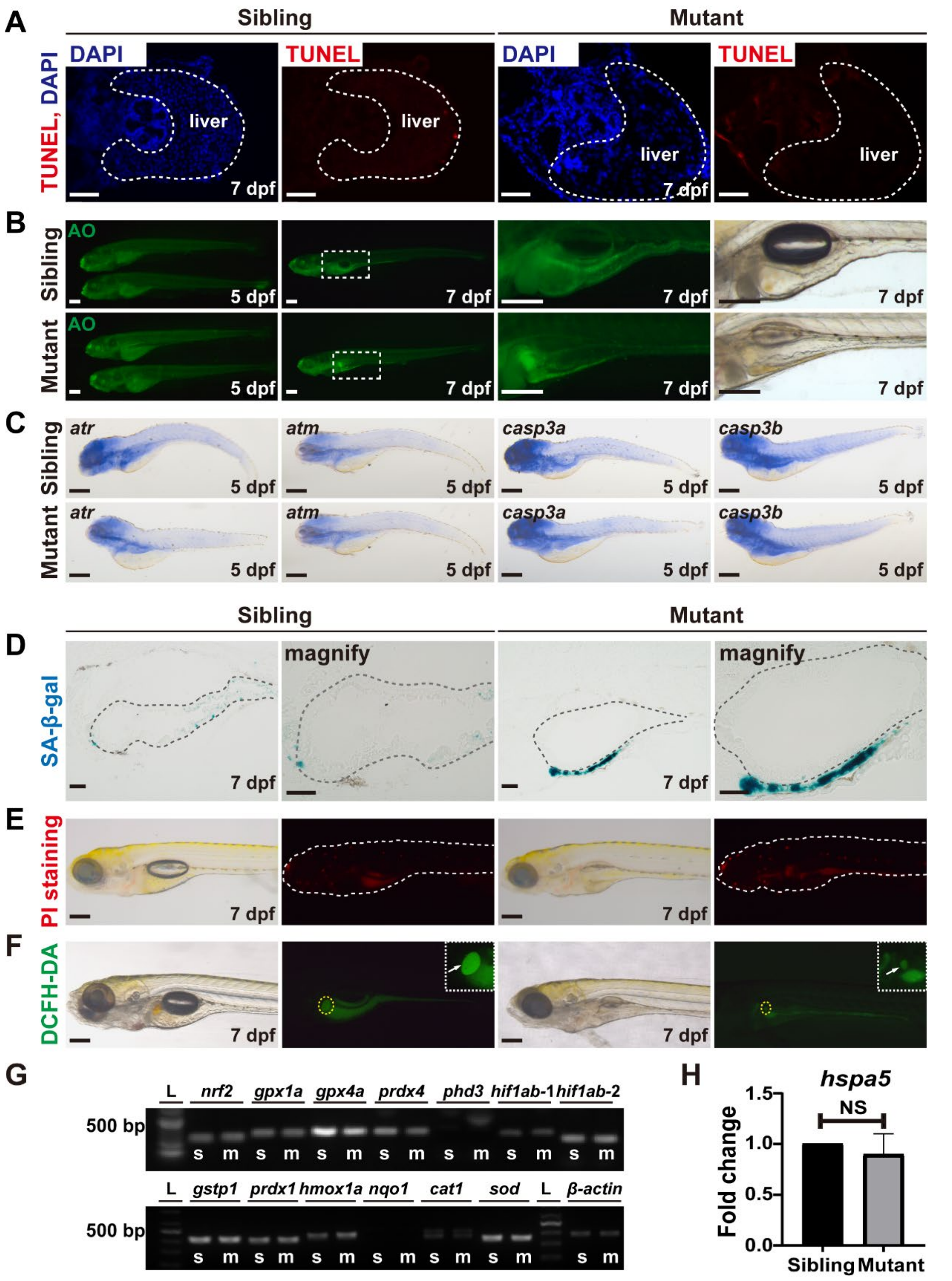

Figure S5. Analysis of apoptosis, senescence, pyroptosis and stresses in *ercc2/xpd* mutants, related to Figure 4

(A) TUNEL assay showed no detectable apoptotic signal in the livers of *ercc2/xpd* mutants and siblings at 7 dpf. Scale bars, 50 μm.

(B) Whole-mount staining with acridine orange (AO) in *ercc2/xpd* mutants and siblings at 5 and 7 dpf. Scale bars, 200 μm.

(C) Whole-mount *in situ* hybridization of DNA damage- and apoptosis-related genes in *ercc2/xpd*

mutants and siblings at 5 dpf. Scale bars, 200 μm.

(D) Senescence-associated β-galactosidase (SA-β-gal) staining showed barely detectable β-galactosidase activity in IECs of *ercc2/xpd* mutants and siblings at 7 dpf. Noted the strong SA-β-gal staining in remained yolk of mutants. Scale bars, 50 μm.

(E) Whole-mount staining with propidium iodide (PI) in *ercc2/xpd* mutants and siblings at 7 dpf. Scale bars, 200 μm.

(F) Whole-mount staining with DCFH-DA in *ercc2/xpd* mutants and siblings at 7 dpf. Inlets show the difference in the size of gallbladder. Scale bars, 200 μm.

(G) Semi-quantitative RT-PCR analysis of ROS related genes in *ercc2/xpd* mutants (m) and siblings (s) at 5 dpf. *β-actin* was used as control. L, DNA ladders.

(H) Real time-PCR analysis of ER stress marker gene *hspa5* in *ercc2/xpd* mutants and siblings at 5 dpf. Data are presented as mean ± SD from three independent biological repeats. Student's t-test, NS, non-significant.

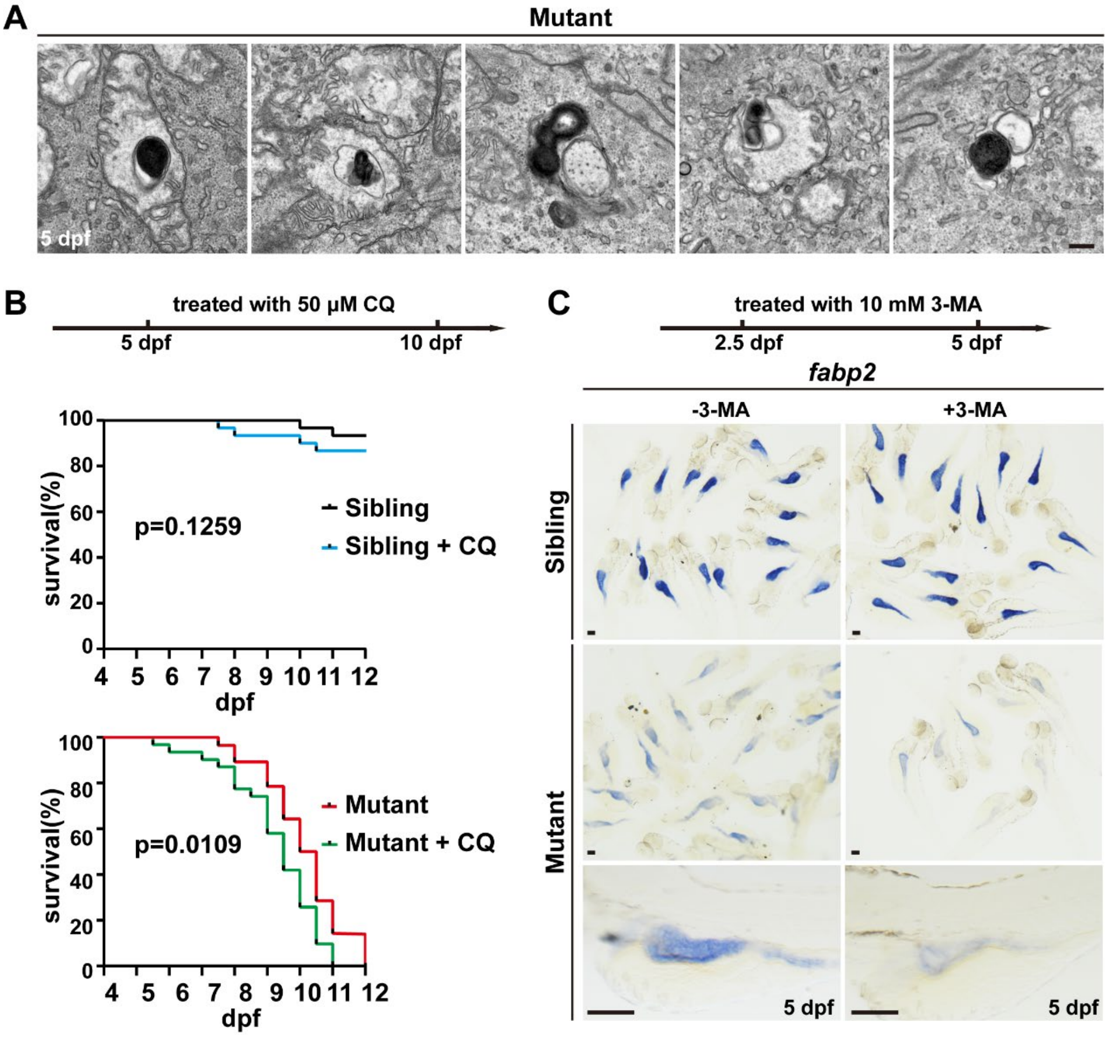

Figure S6. Inhibition of autophagy does not rescue *ercc2/xpd* mutant phenotypes, related to Figure 5

(A) Autophagosome-like structures in IECs of *ercc2/xpd* mutants. Scale bar, 0.2 µm

(B) Survival curves of *ercc2/xpd* mutants and siblings with or without 50 µM chloroquine (CQ) treatment from 5 dpf. Log-rank test, n = 50 larvae each.

(C) Whole-mount *in situ* hybridization of *fabp2* in *ercc2/xpd* mutants and siblings with or without 10 mM 3-MA treatment from 2.5 dpf for 60 h. Scale bars, 200 µm.

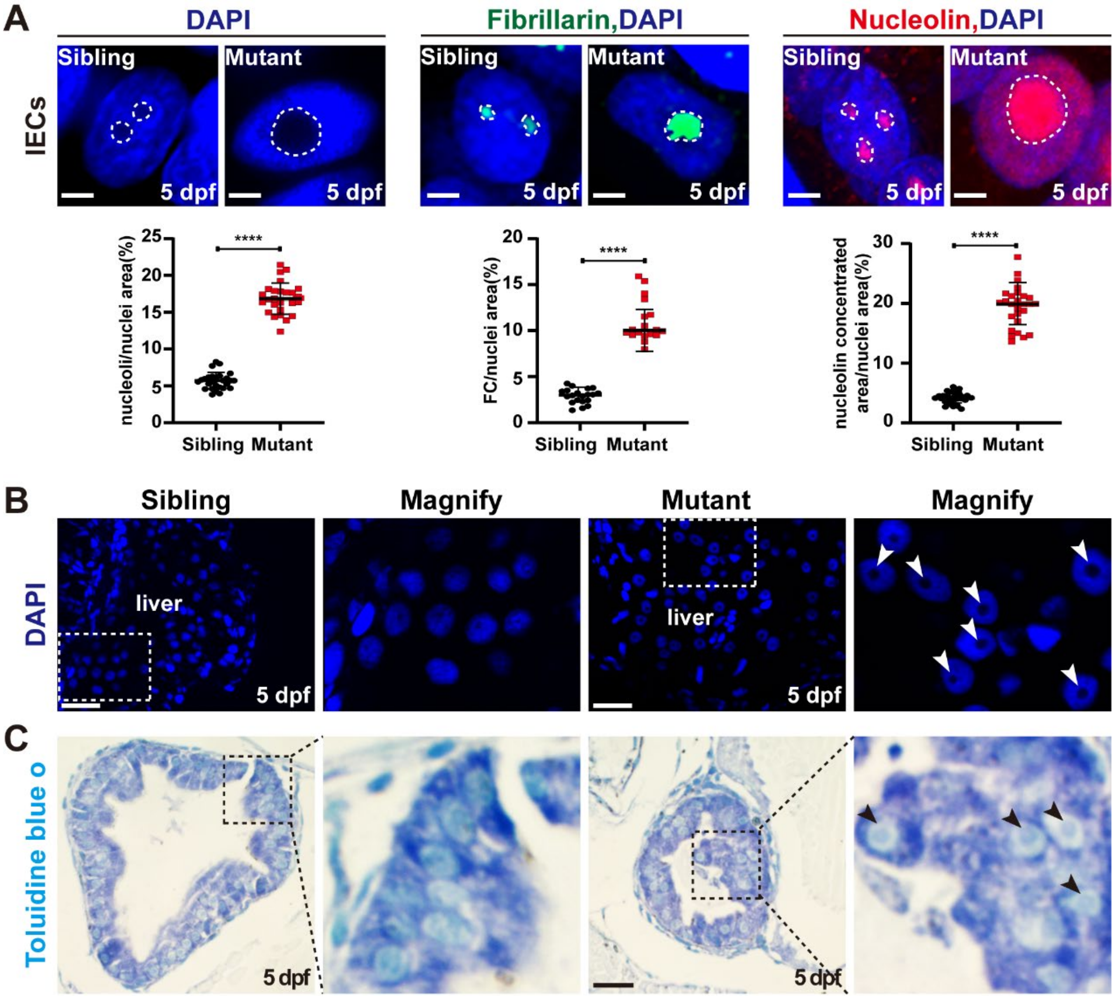

Figure S7. Nucleolar abnormalities in *ercc2/xpd* mutants, related to Figure 6

(A) Quantification of nucleolus size, fibrillar center (FC) and nucleolin concentrated area (outlined area) in the nuclei of mutant and sibling IECs at 5 dpf. Scale bars, 2 μm. Data are presented as mean ± SD, Student's t-test, ****, $P < 0.0001$.

(B) DAPI staining of liver sections in *ercc2/xpd* mutants and siblings at 5 dpf. Areas of dashed boxes are magnified. Arrowheads point to enlarged nucleoli. Scale bars, 20 μm.

(C) Toluidine blue staining of intestinal transverse sections of *ercc2/xpd* mutants and siblings at 5 dpf. Areas of dashed boxes are magnified. Arrowheads point to enlarged nucleoli. Scale bars, 20 μm.

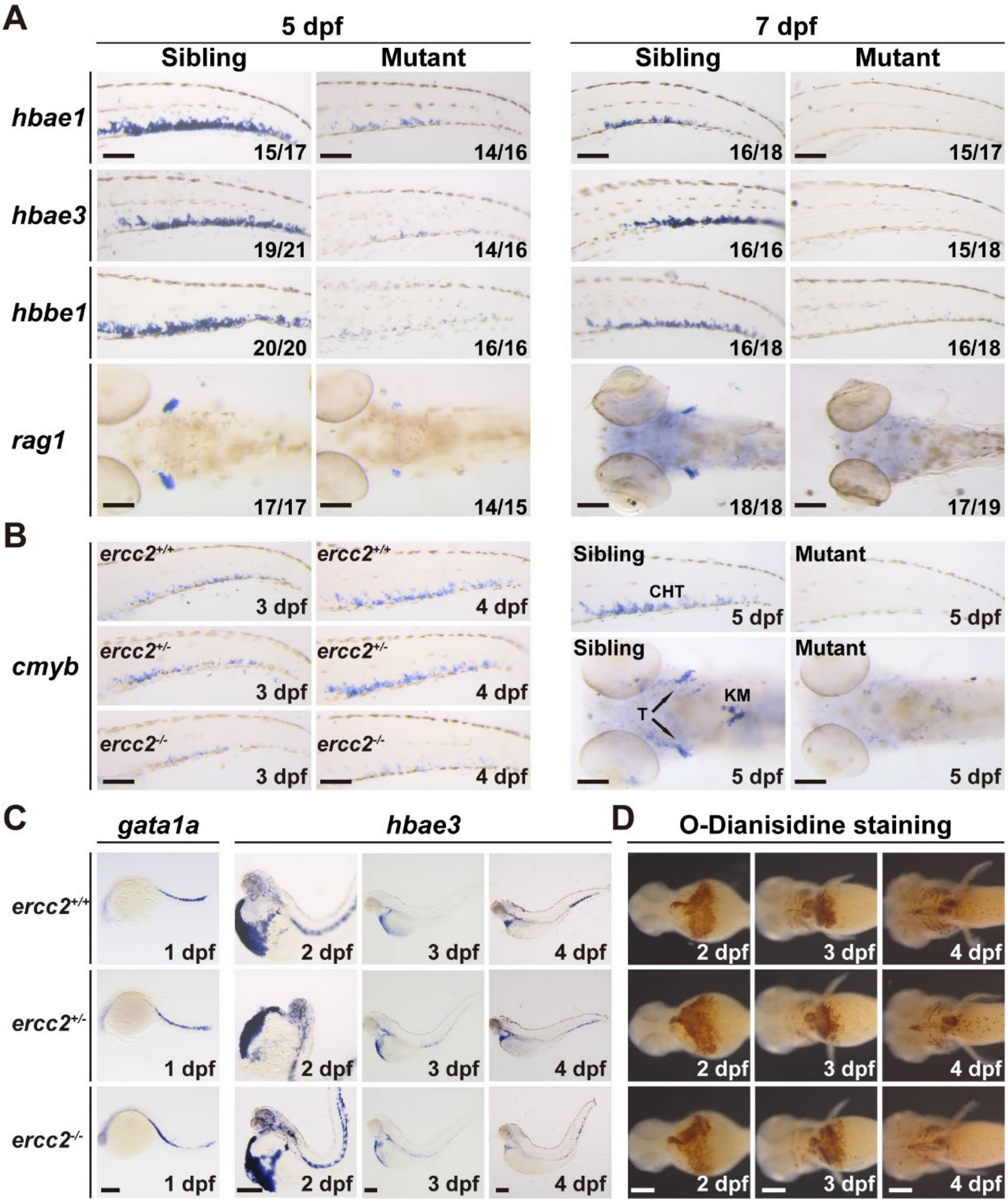

Figure S8. Defective hematopoiesis in *ercc2/xpd* mutants, related to Figure 6 and Figure 7

(A) Whole-mount *in situ* hybridization of hematopoiesis markers in *ercc2/xpd* mutants and siblings at 5 and 7 dpf. Scale bars, 100 μm.

(B) Whole-mount *in situ* hybridization of *cmyb* in *ercc2/xpd* mutants and siblings at the indicated stages. T, thymus; KM, kidney marrow; CHT, caudal hematopoietic tissue. Scale bars, 100 μm.

(C) Whole-mount *in situ* hybridization of *gata1a* and *hbae3* in *ercc2/xpd* homozygous and heterozygous mutants and wild type siblings at the indicated stages. After individual imaging, embryos were subjected to individual genotyping. Scale bars, 200 μm.

(D) Whole-mount O-dianisidine staining in *ercc2/xpd* homozygous and heterozygous mutants and wild type siblings at indicated stages. After individual imaging, embryos were subjected to individual genotyping. Scale bars, 200 μm.

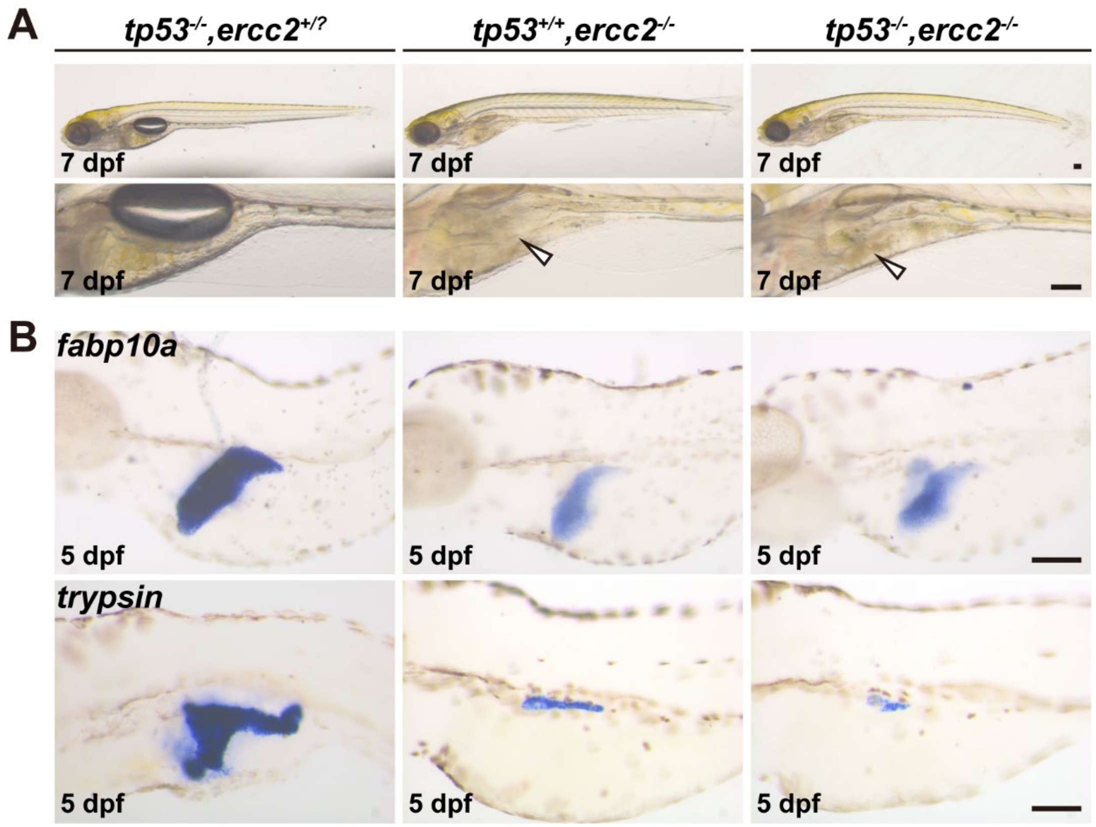

Figure S9. *tp53* mutation does not affect ercc2/xpd mutant phenotypes, related to Figure 7

(A, B) Representative images of gross morphology (A) and whole-mount *in situ* hybridization (B) revealed *tp53* mutation did not affect *ercc2/xpd* mutant abnormalities in digestive organs at the indicated stages. Arrowheads indicate the intestine tubes. Scale bars, 100 μm.

Table S1. List of primers used in this study, related to STAR Methods

Gene	Forward primer (5'-3')	Reverse primer (5'-3')
**WISH probes**		
ercc2/xpd	AGTTCTGCCTGATGAAATCC	ATTCATGCTCCTGTCATTGTTG
anax5	TCAATGCTAACAGCGATGCT	ATGTCCAGCAAGTCCACCTC
gh1	AGGTCTTATGCCTGAGGAAC	AGCCATGCCTGCTTGATATT
vasa	CTCAACCACAAGCATCCATGG	CTGTAGGAAACACCTTTAGGTC
fabp2	TGTTGGGCGGGTGAGATTTA	AAATCCTCTTGGCCTCGACT
fabp6	GAAACCGAATCTCAGGAGG	GTGTCAGAGCAGAAAAGGGT
fabp10a	TACGAGGAGTTTCTCAGAGC	GGTGAAACGCTTCAGATCTTC
trypsin	ATGGTGTTCCATACCAGGTG	TCAAAACTTCCCCGAGCTTA
ins	GTAAGCACTAACCCAGGCA	AAACAGGTGTTTCTGGCATTG
pept1	TCTGATAATGTGCACGCGGA	ATGCTGGATCAGTCACGACG
villin1	ACGCTTCATAGCAACAGAGA	TTTTTGCATGGCACCTCTAC
cdx1b	CGTAAGACACCCAAGCCTAA	TGAAGAGTACCCTGTGTTGG
tfa	AAGTCGTGCATTTTGGAAGC	CATCTTCCCGGGTAATCACA
sepp1b	CTGTCTGCTTTTCCGGCTTT	GTTACTGGCTCTGTACTGTTG
cpa5	AAAGACTATGGACGTGACCC	GGTTGGGACGATTTGATTGG
ela3l	ACTACAGAGTGCTAGTTGGC	TGTCAACCCAGTCAGTGAAA
ela2l	TCCTATTGTGACGAGGGTTG	AGCTCAATCCTGAACCGAAG
gcga	CTGTTAAAATGAAAGGCGTC	AGGGTGAGTTAGCGAAGAGA
sst2	AGCAACTCTTCTCTGTCTGG	GAGTTTTAGCATGCCGTTCTG
foxa3	CTGAATCTGCCCATTCCGA	AGAACTTCCAACATCACCTTT
gata6	AATGAATGGACTCAGCCGAC	ATCAAGGACGTCCCGTATTC
hhex	ACCCGACGCCCTTCTATATT	AGTTTCCCACAACACACGAT
pdx1	CGAAGACTACAGCCAAAACC	CAGTCTTTTGCCACTGGTTG
atr	CTGCCAGTTTTGAGTGTGGC	AACCTTGGCACCAAAGTCCA
atm	GTGGTGCCGGTGTATTCTGA	AGGTGAAGGTTGCCTTGCACTG
casp3a	AAGCCTCAATCCCATGCCTT	GTTTCTTGGCATCAAAGCCCG
casp3b	CAACACCAGAAGCAGGACT	AACCAGGAGCCATTAGCGAC
hbae1	CTCTCTGCCAAAGACAAAGC	AGAGTTTGGAGCACAGTGAA
hbae3	GCTAACCAAGCAACCATGAG	GGCGAATACAGTGTCGCATCAG
hbbe1	ATGGTTGTGTGGACAGACTT	GCTTCAACTCAGAATAGCACA
tp53	TTGTCCCATATGAAGCACCA	TCAGAGTCGCTTCTTCCTTC
**Real Time qPCR primers**		
il1b	CACATCTCGTACTCAAGGAG	GACATTTGACGGACTCGAAG
mmp9	TTTTGCCCTGATCGTGGATA	GGGAAACCCTCCACGTATTT
mmp13a	ATGTGATCCCAATCTGGTCC	GGCCAGAAGCTTTGAATGAG
cxcl8b.1	CTTCAATCTGCACCAGAACT	GAGGTAGAGCTTCACCCTCA
saa	GCCAAAGTTATCAGTGATGG	GGTCTGTATTTGTTGGGGTC
tp53	GGAGGTCTTTTGAGGTGCGT	AAGATTCTTTCACCAAACTACG
ΔN133p53	ATATCCTGGCGAACATTTGG	ACGTCCACCACCATTTGAAC

mdm2	GACAACGAGAAACTGGTAA	AAACATAACCTCCTTCATGGT
hspa5	GATCATCAATGAGCCTACGG	CACTTCAAACACGCCGTTAT
actb1	GATCAAGATCATTGCTCCCC	GGCCATTTAAGGTGGCAACA
**Semi-quantitative RT-PCR primers**		
ercc2/xpd-1	CTACCGAGATCGCTTGTGTA	CCAGAACTCCATGACCCTTT
ercc2/xpd-2	TATCGCCATAAAGCCAGTGT	TGTTTTCAAGGATCCCCTGT
ercc2/xpd-3	CTTTGCGGATAAGCGCTAC	TTAGAAGAGCGTTCAGGCTT
nrf2	GAAATCACACAGAATGGGG	CAACTCTGGGAGTGACAGC
gpx1a	CAGTTCGGGCACCAGGAGAA	CCTCTTGAATGGTTCCCCGT
gpx4a	GCAGTCATGCGTTTCTTAGG	GGAATTAGTTCCTGGTTCCTGA
prdx4	AGTTTACTCATCTGGCCTGG	AGGGTCGGGGATAATCGTAT
phd3	TTTGAATGCACCAGCCTCAG	ACAAGCCACCATTGCCTTTGA
hif1ab-1	ACTACGTTCTCAGTGGCATT	TGACATCATTGTAGAGGGGC
hif1ab-2	ACTACGTTCTCAGTGGCATT	ATGCCACTGTTGAGTATCGG
gstp1	CTACCAGGAATATGAGACCG	CATTGAGCACCAGTTAAGGC
prdx1	GTCTGACTACAAAGGGAAGT	GGTTTCATCAATGGAGCGAC
hmox1a	GAGACTGAGAGAGATTGGC	CTAATGCGAACTGAAGGGCT
nqo1	CATTCGACTGCAAAGTGTCA	CTTCTGCGATCAAGCTGAAAG
cat1	CTTCATCCAGAAACGCATGG	TATGCAAAGGCCCCCATTTT
sod1	CCGTTTGTGTGCTTAAAGGC	TCCTCCTTCTCATGAATCACCAT
actb1	CAGCCTTCCTTCCTGGGTAT	GCCATACAGAGCAGAAGCCA
**Northern blot probes**		
5'ETS	CCGGTCTACCTCGAAAGTC	CGAGCAGAGTGGTAGAGGAAG
ITS1	CTCGGAAAACGGTGAACCTG	GTGTTCGTTTCAGGGTCCG
**Genotyping primer**		
ercc2/xpd	GCTTTGAATCAGGCACGAC	AGTATCTAAGTGCGAGCGTTG
